# YAP1 reactivation in cardiomyocytes following ECM remodelling contributes to the development of contractile force and sarcomere maturation

**DOI:** 10.1038/s41420-025-02793-2

**Published:** 2025-11-10

**Authors:** Vladimir Vinarsky, Stefania Pagliari, Bacel Aldabash, Fabiana Martino, Cristina Mazzotti, Katerina Jirakova, Zuzana Garlikova, Enrico Di Iuri, Daniel Kytyr, Patrizia Benzoni, Martina Arici, Alessia Metallo, Kira Zeevaert, Wolfgang Wagner, Marcella Rocchetti, Giancarlo Forte

**Affiliations:** 1 International Clinical Research Center (ICRC), St Anne’s University Hospital, Brno, Czech Republic; 2 Faculty of Medicine, Masaryk University, Brno, Czech Republic; 3 School of Cardiovascular and Metabolic Medicine and Sciences, King’s College London, London, UK; 4 National Heart and Lung Institute, Imperial College London, London, UK; 5 Institute of Physiology, University of Bern, Bern, Switzerland; 6 Institute of Theoretical and Applied Mechanics of the Czech Academy of Sciences, Prague, Czech Republic; 7 Università degli Studi di Milano, Milan, Italy; 8 Università degli Studi di Milano-Bicocca, Dept of Biotechnology and Biosciences, Milan, Italy; 9 Institute for Stem Cell Biology, RWTH Aachen University Medical School, Aachen, Germany; 10 Helmholtz-Institute for Biomedical Engineering, RWTH Aachen University Medical Faculty, Aachen, Germany

**Keywords:** Extracellular signalling molecules, Embryonic stem cells, Cardiomyopathies

## Abstract

Cardiac diseases are fueled by extracellular matrix (ECM) remodelling. Together with the altered ECM chemical composition, the mechanical turmoil associated with ECM maladaptive remodelling in the pathological heart drives the shuttling of Yes Associated Protein 1 (YAP1) into cardiomyocyte (CM) nuclei that results either in cell cycle re-entry or cardiomyocyte hypertrophy. The mechanism of YAP1 reactivation and factors driving qualitatively different cellular outcomes is not well understood. Here we employed mechanical actuation as a proxy reproducing ECM remodelling in vitro to trigger YAP1 nuclear shuttling in contractile cardiomyocytes derived from human embryonic and induced pluripotent stem cells (hPSCs). By using hPSC lines in which YAP1 expression has been genetically depleted, super-resolution microscopy and electrophysiological measurements, we show that ECM-triggered nuclear presence of endogenous YAP1 contributes to cardiomyocyte maturation, participates in the formation and alignment of myofibrils, as well as in the maturation of their electrophysiological properties and calcium dynamics. We eventually exploit engineered heart tissues (EHTs) to demonstrate that the net effect of YAP1 deficiency in cardiomyocytes is the inability to respond to physiological stimuli by compensatory growth that results in reduced force development. These results suggest that the re-activation of endogenous YAP1 following ECM maladaptive remodelling promotes cardiomyocyte contractility by restructuring the sarcomere apparatus and the maturation of electrophysiological properties via transcriptionally dependent and independent mechanisms.

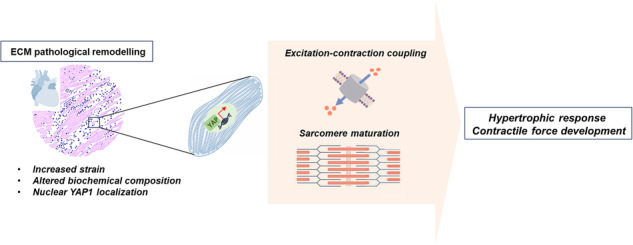

## Introduction

Cardiac pathologies are characterized by ECM remodelling (expansion of cardiac fibroblasts, deposition of stiffer ECM containing higher amounts of fibronectin (FN)) that reduces organ functionality [1]. Independently of the aetiology of the disease, cardiac remodelling causes major shift in cardiomyocyte (CM) contractile function. Cells in direct contact with the remodelled ECM (at the infarction border zone, for example) re-express the transcriptional co-factor containing WW domain(s) and PDZ binding motif (PDZbm) Yes Associated Protein 1 [2, 3]. YAP1 is not commonly detected in the nuclei of CMs in adult healthy heart [4]; its nuclear localization in CMs is accompanied by CM hypertrophy [5]. Several seminal studies demonstrated that removing YAP1 inhibitory pathway Hippo or re-expressing ectopically the constitutively active YAP1 restarts proliferation of adult cardiomyocytes [6], disrupts sarcomere structure but does not lead to CM hypertrophy [7, 8].

YAP1 transcriptional activity relies on its nuclear entry/exclusion. Mechanosensitive Hippo signalling pathway is the predominant inhibitor of YAP1 nuclear entry; LATS1/2 mediated phosphorylation of YAP1 on serine 127 (S127) leads to 14-3-3 binding, cytoplasmic retention and degradation [9]. Inhibition of Hippo signalling by biochemical or mechanical stimulation dephosphorylates YAP1 and enables nuclear entry. In addition to Hippo pathway, F-Actin assembly, Rho, AMPK, and Erbb4 activation [10, 11] promote YAP1 nuclear localization independently of Hippo. The strong positive effect of the S127 residue on nuclear YAP1 localization led to development of the constitutively nuclear, active version of YAP1 (YAP-S127A) and its derivatives (S5A, S8A) which have been used in the majority of studies on YAP1 function; especially in the context of heart regeneration [6, 8, 12, 13]. The nuclear localization of YAP1 depends on intact c-terminal motif of YAP1 that binds PDZ domain of tight junctions and cytoskeletal proteins (PDZbm) [14].

The transcriptional activity of YAP1 is executed through the WW domain mediated interaction with multiple transcriptional factors (TFs). TEF family transcription factors (TEADs), RUNX1/2, Erbb4, TBX5, SMAD, p73 and others have been shown to interact with YAP1 and promote various cellular outcomes including development, proliferation, cell survival, cell polarity, and apoptosis [14–17]. YAP1-TEAD mediated transcription has the widest impact on gene expression and has been studied extensively in a range of cell types. TEAD binding M-CAT (ATTCC) sequence was first identified in promotors of oncogenesis related YAP1-TEAD targets (CCN1, CCN2) but is present also in the promoters of cardiac genes (MYH7, NPPB, ANKRD1) [18, 19]. Another important layer of transcriptional regulation is YAP1-TEAD binding to enhancers (>10 kbp from TSS) [19–21]. YAP1 binding to enhancers constitutes the majority of YAP1 binding to the genome and further broadens the transcriptional effect of YAP1.

The volume of observations concerning the effects of cytoplasmic or membrane bound YAP1 is considerably smaller. Cytoplasmic YAP1 mediated cell polarization, observed both in the development of epithelia [22] and hematopoietic system [23], requires intact PDZbm domain. Interestingly, mutually exclusive roles of cytoplasmic/nuclear YAP1 were observed; ectopic YAP1 variants forced to cytoplasm (PDZbm deficient YAP1 or YAP-S127D) promote migration of HUVECs, but nuclear YAP1 S127A reduces it [24]. Of note, the role of cytoplasmic/membrane-bound YAP1 has been connected with the activity of Cdc42, small GTPase that participates on the regulation of cardiac development [25, 26] and hypertrophy [27]. Although multiple sarcomere and Z-disc proteins contain PDZ domains, no direct interaction between YAP1 and sarcomere proteins have been detected so far.

The activity of Hippo-YAP1 pathway during cardio genesis is multiphasic. Initially, high transcriptional activity of YAP1 protects pluripotency in stem cells [28]. Later, its expression is repressed to enable mesoderm specification [29, 30] to be again re-activated to drive the proliferation of embryonic cardiomyocyte progenitors [31]. The absence of either YAP1 or TEAD at the progenitor expansion stage leads to hypoplastic heart and embryonic death [6, 32]. In adult heart, Hippo mediates the nuclear exclusion of YAP1 to stop cardiomyocyte proliferation and prevent cardiomegaly [33]. Still, finely tuned YAP1 activity is required for adult heart function. For example, while the perinatal deletion of TEAD1 [34] or YAP1 in cardiomyocytes leads to dilated cardiomyopathy after increased load or injury [3, 5], so does the constitutive repression of Hippo pathway or TEAD overexpression after pressure overload [35, 36]. Significant efforts are being made to identify and exploit the subsets of YAP1 targets driving cardiomyocyte proliferation since the discovery that YAP1 sits at the crossroad of a handful of microRNAs being able to reactivate adult CM proliferation [37, 38].

Here we set up a model to study the ECM mediated activation of endogenous YAP1 based on mechanical actuation to mimic YAP1 nuclear shuttling in human embryonic and induced pluripotent stem cells (hESCs and hiPSCs, respectively). We next took advantage of CRISPR-Cas9-generated YAP1-KO hESCs and hiPSCs to investigate the role of endogenous YAP1 on gene expression and function of cardiomyocytes. We show that YAP1 deficiency disrupts the development and maturation of CMs contractile and excitation-contraction (EC) coupling apparatus, a phenomenon that can be partially rescued by YAP1 re-expression in beating CMs that is not dependent on YAP1-TEAD activity. In addition, we highlight how YAP1 absence abolishes the compensatory response to biochemical and mechanical stimulation.

Finally, we demonstrate that the complex effects of YAP1 deficiency on the excitation and contraction apparatus determine a significant reduction in the force generated by engineered heart tissues (EHTs). This work shows previously unattended observations of YAP1 role in CM maturation and physiology, which could be leveraged for therapeutic use in heart failure.

## Results

### YAP1 shuttles to the nucleus following ECM remodelling to determine cardiomyocyte compensatory growth response

Cardiomyocytes (CMs) at the myocardial infarction (MI) border zone experience unique environmental conditions due to the altered ECM composition and increased mechanical strain [39, 40].

We set at establishing a reductionist in vitro model to break down the contribution of ECM chemical composition and mechanical stress on YAP1 reactivation in cardiomyocytes. We differentiated YAP1-KO hESCs and their isogenic control line (hereafter referred to as WT) into spontaneously beating CMs according to an established protocol [41] for 15 days. Next, we exposed hESC-derived cardiomyocytes cultured onto 10 kPa polydimethylsiloxane (PDMS) to either 1 μg/ml or 10 μg/ml of fibronectin coating, or mechanical actuation (120% static stretch for 24 h).

**Fig. 1:**
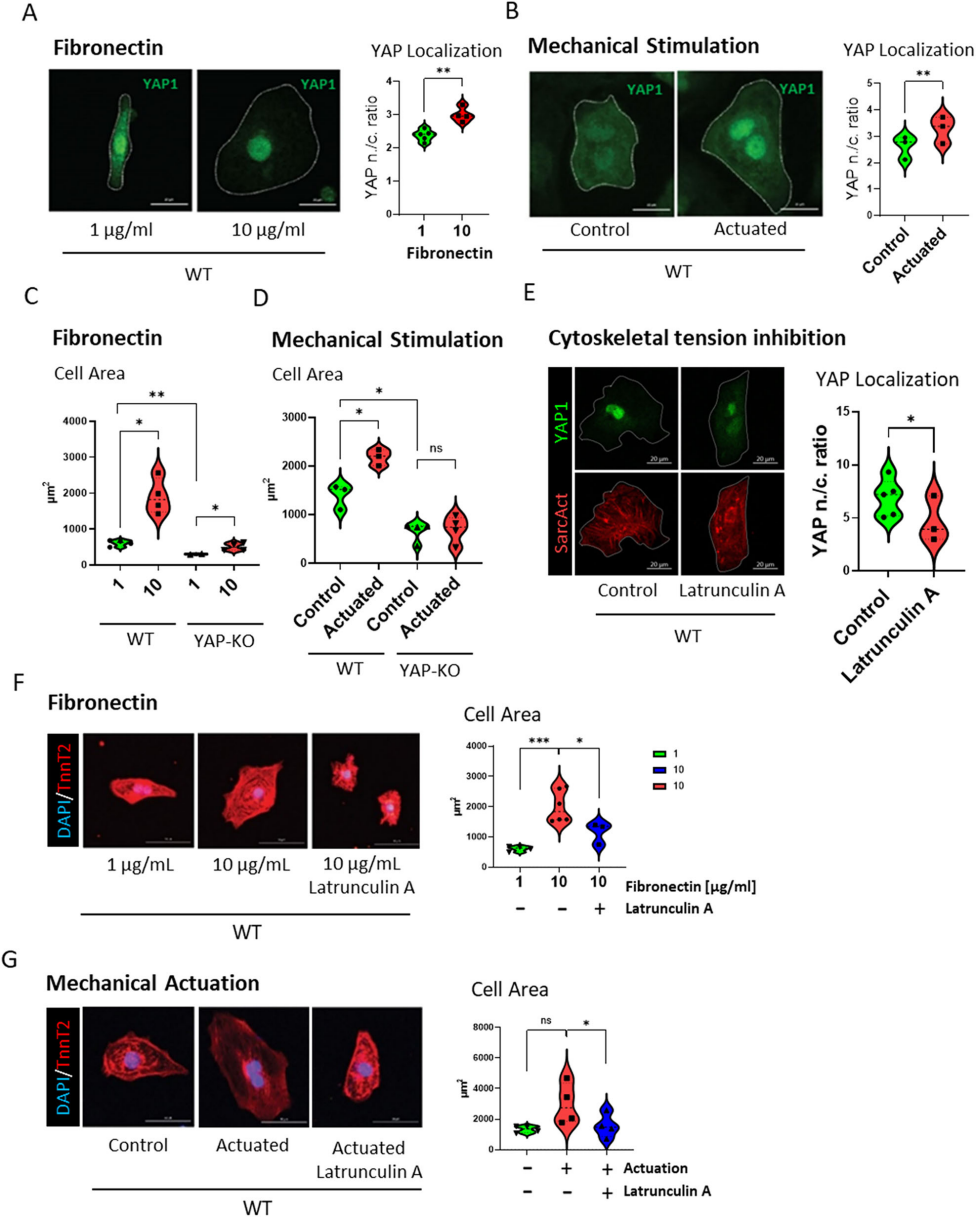
ECM remodelling induces cardiomyocyte area increase via cytoskeleton-mediated YAP1 nuclear shuttling. Representative images and violin plot representation of nuclear translocation of YAP1 in hESC-WT cardiomyocytes in response to fibronectin (left) (*P* = 0.0041, *N* = 4, *n*(FN1) = 129, *n*(FN10) = 135, utt) (**A**) and mechanical actuation (*P* = 0.0077, *N* = 3, *n*(Control) = 81, *n*(Actuated) = 94, ptt) (**B**). Violin plot representation of projected cell area of WT (*P* = 0.0122, *N* = 4, *n*(FN1) = 191, *n*(FN10) = 239, utt) and YAP1-KO (*P* = 0.0283, *N* = 2, n(FN1) = 265, *n*(FN10) = 579, utt) CMs in response to fibronectin (**C**) and mechanical actuation (WT: *P* = 0.0067, *N* = 3, *n*(Control) = 81, *n*(Actuated) = 94, ptt) (YAP-KO: *P* = 0.5988, *N* = 4, *n*(Control) = 302, n(Actuated) = 264, ptt) (**D**). Representative images of YAP1 localization and violin plot quantification of nuclear/cytoplasmic (n/c) ratio of YAP1 intensity in untreated (Control) and latrunculin A (250 nM, 24 h) treated WT cells (*P* = 0.0186, *N* = 3, *n*(Control) = 217, *n*(latrunculin A) = 187, ptt) (**E**). Attenuation of cellular response in presence of latrunculin A mediated cytoskeletal tension inhibition in response to fibronectin (**F**), mechanical actuation (**G**) in hESC-WT CMs. Representative confocal images of hESC-WT CMs stained for cardiac troponin-T (red) and DAPI (blue), images (**F**, **G** left), quantification of projected cell area upon fibronectin deposition (FN10 vs FN10 + LatA, *P* = 0.0306, *N* = 3, *n*(FN10) = 342, *n*(FN10 + LatA) = 192, utt) (**F**, right) and mechanical actuation (Actuated vs Actuated + LatA, *P* = 0.0232, *N* = 4, *n*(Actuated) = 277, *n*(Actuated + LatA) = 267, ptt) (**G**, right). Statistics: utt unpaired *t* test, ptt paired *t* test, ns: *P* > 0.05, *P < 0.05, **P < 0.01, ***P < 0.001, ****P < 0.0001.

Increased fibronectin deposition induced the nuclear localization of YAP1 in CMs (nuclear/cytoplasm ratio 2.380 ± 0.1719 in 1 μg/ml vs 3.002 ± 0.1096 in 10 μg/ml, unpaired *t* test, *P* = 0.0041) (Fig. 1A). Static stretch also induced the translocation of YAP1 into CM nucleus (from 2.617 ± 0.4401 in control vs 3.278 ± 0.5072 actuated, paired *t* test, *P* = 0.0077) (Fig. 1B). These results indicated that both altered ECM composition and increased mechanical stress contribute to YAP1 reactivation in cardiomyocytes.

Having established the ability of our setup to induce the nuclear localization of YAP1 in WT hESC-CMs we meant to validate the model by monitoring the effects of ECM-mediated YAP1 nuclear reactivation on hESC-CMs. Since YAP1 re-expression has been previously associated with cardiomyocyte hypertrophic response [4], we employed CMs differentiated from hESCs in which YAP1 expression has been genetically and stably depleted by CRISPR/Cas9 (YAP1-KO CMs) [28].

We first confirmed that YAP1-KO CMs were significantly smaller than the control (WT) CMs (YAP1-KO: 304.2 ± 13.54 µm^2^ vs. WT: 612.6 ± 84.11 µm^2^, unpaired *t* test, *P* = 0.0041) on the lower concentration of fibronectin (1 μg/ml). Interestingly, fibronectin accumulation (10 μg/ml) increased the projected area of both WT and YAP1-KO CMs (from 612.6 ± 84.11 µm^2^ to 1909 ± 493.9 µm^2^, unpaired *t* test *P* = 0.0122 in WT CMs, and from 304.2 ± 13.54 µm^2^ to 506 ± 104.2 µm^2^, unpaired *t* test *P* = 0.0283 in YAP1-KO) (Fig. 1C). In all conditions YAP1-KO cells were significantly smaller than the WT counterparts.

Static stretch (120%, 24 h, a recognized model of volume overload) increased cell area in WT CMs (control: 1402 ± 258.4 µm^2^ vs. actuated: 2187 ± 165.9 µm^2^, paired *t* test, *P* = 0.0067); this response was abolished in YAP1 depleted CMs (control: 663 ± 203.5 µm^2^ vs. actuated: 702.3 ± 281.1 µm^2^, paired *t* test, *P* = 0.5988) (Fig. 1D). These data indicated that the hypertrophic response induced by mechanical stress requires YAP1 shuttling, while fibronectin accumulation during ECM remodelling might be able to induce changes in cell area independently of YAP1.

To further confirm that the hypertrophic response dependent on mechano-regulated YAP1 nuclear shuttling, we inhibited cytoskeleton-built intracellular tension using a pharmacological inhibitor of F-actin cytoskeleton polymerization [42, 43]. First, we confirmed that latrunculin A reduced YAP1 nuclear localization (from 6.577 ± 2.390 to 4.674 ± 2.147 paired *t* test, *P* = 0.0186) (Fig. 1E). In addition, we observed a significant disruption of the myofibrillar structures (Supplementary Fig. 1A) suggesting the relaxation of intracellular tension. Next, we observed that the inhibition of F-actin polymerization and YAP1 shuttling abolished cell growth induced by fibronectin deposition (from 2011 ± 530 µm^2^ to 1163 ± 360,2 µm^2^, unpaired *t* test, *P* = 0.0306) (Fig. 1F) or increased strain conditions (from 2988 ± 1341 µm^2^ to 1554 ± 776,4 µm^2^, paired *t* test, *P* = 0.0232)(Fig. 1G). As the effect of latrunculin A is broader than only YAP1 nuclear shuttling we investigated the effect of a YAP1 activity inhibition using a YAP1-TEAD inhibitor verteporfin. Verteporfin treatment (0.05 µM, 24 h) showed a tendency to reduce WT CMs area (from 2413 ± 898.3 µm^2^ to 1969 ± 1015 µm^2^ paired *t* test, *P* = 0.168) without dramatic changes of sarcomere structures (Supplementary Fig. 1B).

In summary, these data indicated that both fibronectin accumulation and mechanical stress, two phenomena associated with ECM remodelling, trigger YAP1 nuclear shuttling in hESC-CMs and promote their size growth. Fibronectin accumulation can also induce the same phenotype independently of YAP1.

### Dissecting YAP1 transcriptional activity in hESC-CMs

**Fig. 2:**
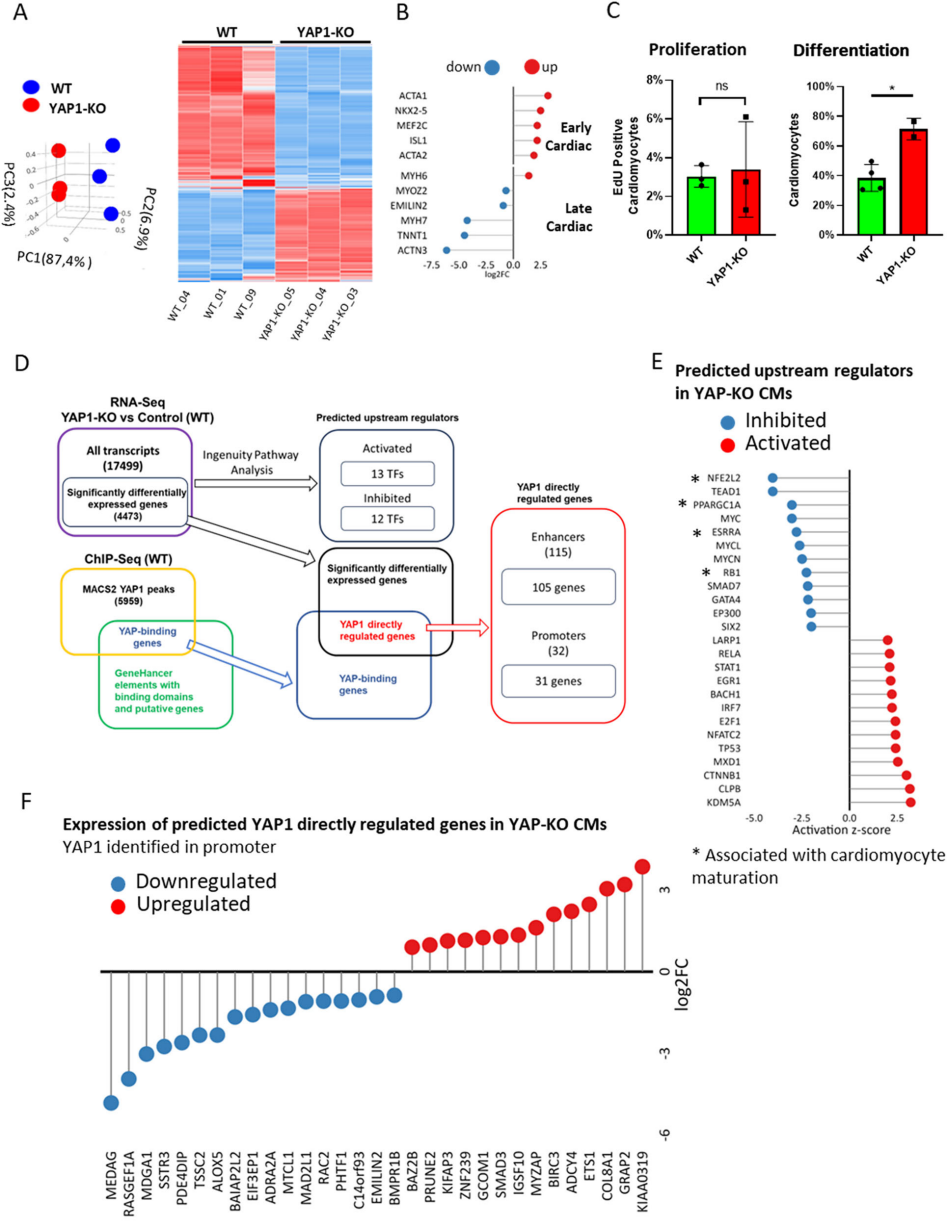
YAP1 transcriptional activity in hESC-CMs. Principal component analysis (PCA) (left) and heatmap representation (right) of the differentially expressed genes (DEG) in WT and YAP1-KO hESC-CMs as identified by RNA-sequencing (day 15 of differentiation, *n* = 3) (**A**). Lollipop plot representation of differential gene expression of key CM maturation genes in YAP1-KO compared to WT hESC-CMs (*n* = 3) (**B**). Bar plot representation of percentage of EdU-positive (left) and troponin-T positive (right) YAP1-KO and WT hESC-CMs (*N* = 3) (**C**). Scheme of analysis of gene expression data (**D**). Lollipop representation of IPA predicted upstream regulators based on differential expression (**E**). Lollipop plot representation of gene expression of the differentially regulated genes with predicted YAP1 binding based on GeneHancer database (**F**).

Since YAP1 acts as a transcriptional co-factor in numerous cell types, we set at dissecting the transcriptional profile of hESC-CMs in which YAP1 has been reactivated by ECM remodelling. For this purpose, YAP1-KO hESCs and their isogenic control line were differentiated into spontaneously beating CMs for 15 days. At this point, total RNA was harvested and analysed by RNA sequencing. Principal component analysis (PCA) of the RNA-seq data indicated that YAP1 absence consistently and reproducibly determined a shift in hESC-CMs transcriptional landscape away from the WT. One quarter (4473 out of 17,499) of all detected genes were differentially regulated in YAP1-KO CMs (fold change >1.5 and adjusted *p* value < 0.05) (Fig. 2A; Supplementary Table 1). Importantly, genes involved in late CM maturation (MYOZ2, EMILIN2, MYH7, TNNT1, ACTN3) were significantly downregulated in YAP1-KO cells, which expressed significantly higher levels of early cardiac commitment markers (ACTA1, NKX2-5, MEF2C, ISL1, ACTA2) (Fig. 2B).

Altogether these results indicated that, although hESCs in which YAP1 has been genetically depleted retain the ability to differentiate into early contractile cells, the absence of the protein affects their maturation. This result could not be explained by a difference in the proliferation of less mature YAP1-KO hESC-CMs (3.0% ± 0.6% in WT and 3.4% ± 2.5% in YAP1-KO) (Fig. 2C, left), but rather by their differentiation efficiency (38,5% ± 9.06% in WT vs 71.44% ± 7.30% in YAP1-KO) (Fig. 2C, right).

To gain an insight into upstream regulatory transcription factors involved in changes of gene expression in YAP-KO depleted hESC-CMs compared to WT, we used Ingenuity Pathway Analysis (IPA). Based on our RNA-seq data we identified 12 inhibited and 13 activated transcription factors using |z-activation score| = 2 as a cutoff (scheme in Fig. 2D) in YAP1 depleted cardiomyocytes. IPA predicted inhibition of TEAD1 and MYC activity in YAP1-KO CM’s which is in line with previous observations [20, 44]. Among the other predicted upstream regulators, we found NFE2L2 (NRF2), PPARGCC1A, ESSRA, and RB1 (labelled by asterisks in Fig. 2E) which have been described to promote cardiomyocyte maturation in cardiomyocytes through block of cell cycle progression, structural organization, and metabolic changes [45–48].

To understand direct involvement of endogenous YAP1 protein on transcription in WT contractile cells we analysed YAP1 DNA binding activity by chromatin immunoprecipitation (ChIP) followed by sequencing. See Supplementary Table 2 for the complete list of YAP1 binding sites in hESC-CMs. Next, we integrated the data obtained by RNA-seq in YAP1-KO hESC-CMs with those generated by ChIP-seq analysis in the WT CMs to identify YAP1 directly regulated genes as those genes which significantly changed their expression and YAP1 was physically bound to enhancers or promoters annotated in GeneHancer (*p* value < 0.05 with 1000 permutation) [44]. Through this strategy, we identified its presence on 115 enhancers which regulated 105 genes. See Supplementary Table 3 for the full list of GeneHancer identified enhancer-gene pairs with the method used for inferring the connection. Thirty-six of these genes were previously described to be regulated by YAP1 at enhancers [20]. In addition, YAP was bound to 31 promoters of 30 differentially expressed genes (Fig. 2F). While we observed repression of a handful genes involved in cardiomyocyte differentiation through BMP axis (BMPR1B) [49], survival (BIRC3) [50] sarcomere organization (RAC2) [51], and hypertension (ADRA2) [52, 53]; in YAP1-KO CMs, we did not observe binding to previously described non-CM targets such as CTGF, CYR61, and AXL or to CM specific hypertrophy associated targets containing TEAD consensus sequence such as MYH7 or NFAT. We would reason that our samples of unstressed confluent beating cultures did contain very low level of YAP1 activity, unless it is unleashed upon stimulation. Taken together, our data suggest that YAP1 activity in mechanically non-stimulated conditions, while important for cardiomyocyte development and maturation, acts in trans without involvement of previously identified canonical target genes of activated YAP1.

### YAP1 expression promotes sarcomere maturation in hESC-CMs

The hypertrophy like effects of YAP1 reactivation downstream of ECM remodelling (shown in Fig. 1) in hESC-CMs - which to some extent recapitulate foetal CM development - lead us to focus on the expression of gene subsets essential during heart development. In the absence of a specific annotation among the YAP1 bona fide targets that could account for heart development categories, we zoomed out and looked for indirect targets in the RNA-seq dataset of spontaneously contracting hESC-CMs.

**Fig. 3:**
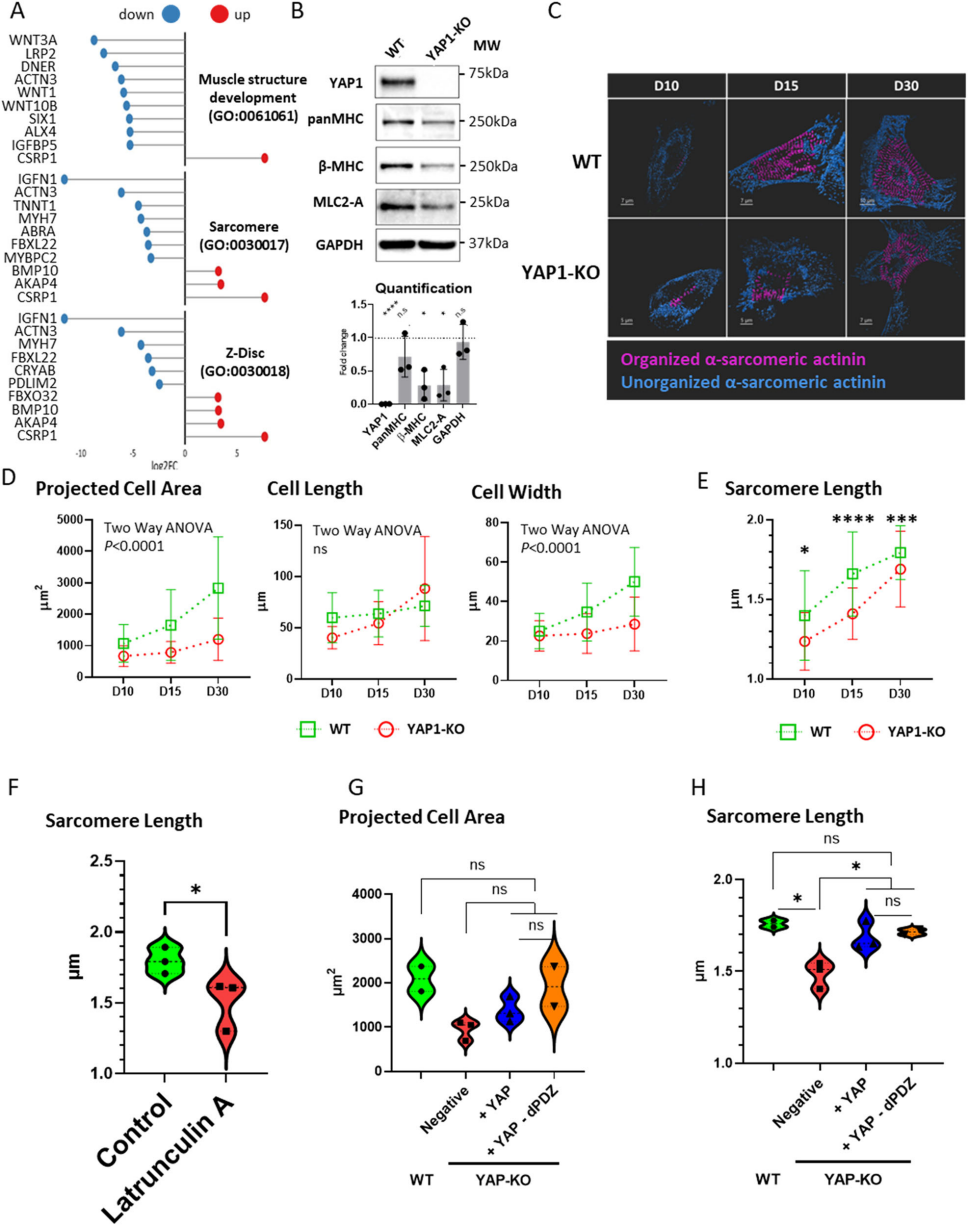
YAP1 is instrumental for sarcomere assembly and maturation in hESC-CMs. Lollipop plot representation of differential gene expression of indicated gene ontology (GO) categories in YAP1-KO compared to WT hESC-CMs (*n* = 3) (**A**). Western blot analysis of the indicated sarcomere proteins in YAP1-KO and WT CMs quantification on bottom; GAPDH was used for normalization (**B**). Characterization of changes in morphology and sarcomere length in WT and YAP1-KO CMs at days 10, 15, and 30 of differentiation. Representative segmentation of α-sarcomeric actinin into organized (magenta) and disorganized (blue) structures (**C**). Morphometry of projected cell area, length, width (**D**), and sarcomere length (**E**). Statistics: Two-way ANOVA, Interaction: *P* < 0.0001. HESC-WT CMs at day 16 were untreated (Control) or incubated with latrunculin A. Analysis of sarcomere length sarcomere length (*P* = 0.046, *N* = 3, *n*(Control) = 62, n(Latrunculin A) = 53, ptt) (**F**). Violin plot representation of WT and YAP1 deficient hESC-CMs (YAP-KO Negative) with re-expressed full length YAP (+YAP) or PDZ domain binding motif deficient YAP (+YAP-dPDZ) projected cell area (**G**) and sarcomere length (Negative vs +YAP, *P* = 0.0305, *N* = 3, *n*(Negative) = 161, *n*( + YAP) = 74, utt), (Negative vs +YAP-dPDZ, *P* = 0.0245, *N* = 2, *n*(Negative) = 161, *n*(+YAP-dPDZ) = 78, utt) (**H**). The data are shown as mean ± S.D. Statistics: utt unpaired *t* test, ptt paired *t* test, ns *P* > 0.05, *P < 0.05, **P < 0.01, ***P < 0.001.

Among the genes consistently dysregulated in YAP1-depleted hESC-CMs, we identified genes involved in the development of muscle structure, sarcomere, and Z-disc. Namely we confirmed the significant down-regulation of the cardiac mesoderm specification WNT3A [29, 30] and observed a similar tendency in sarcomere and Z-disc associated genes (ACTN3, TNNT1, MYH7, CRYAB, IGFN1), genes connected with sarcomere turnover (FBXL22), actin binding (PDLIM2, LMOD2), and Rho-activated transcription (ABRA) (Fig. 3A; Supplementary Fig. 2) which point to the disruption of sarcomere structure development and maturation.

Indeed, we noted that bulk cultures of YAP1-KO CMs consistently contained less sarcomere proteins (Fig. 3B). This fact, together with the observed differences in cell size and response to hypertrophic stimuli described in Fig. 1, lead us to quantify differences in the morphology and sarcomere maturation using confocal and super-resolution microscopy throughout the maturation of hESCs-derived cardiomyocytes (Fig. 3C).

We compared projected cell area, morphology, sarcomere organization and length from the onset of beating (day 10) to day 30 of differentiation. The projected area of WT CMs increased nearly threefold from 1068 ± 601 µm^2^ at day 10 to 2831 ± 1625 µm^2^ at day 30 (*P* < 0.001, two-way ANOVA), with major contribution coming from the cardiomyocyte width (from 25 ± 8.9 µm to 50 ± 17.4 µm). YAP1-KO CMs growth was instead limited to less than twofold (*P* < 0.0001, two-way ANOVA) with no significant change in CM width (from 23 ± 8 µm at day 10 to 29 ± 13.6 µm at day 30) (Fig. 3D). These data indicate that YAP1 is involved in sarcomere assembly and maturation.

Together with increased cell area, hypertrophic cardiomyocytes are characterized by augmented sarcomere assembly. We monitored sarcomere length in WT and YAP1-KO cardiomyocytes during differentiation and observed that sarcomere length in YAP1 depleted cells was markedly shorter than YAP1-KO cells from the beginning of myofibril assembly (at day 10) (WT: 1.39 ± 0.281 µm vs. YAP1-KO: 1.24 ± 0.18 µm; multiple comparison *t*-test Q = 1%, *P* = 0.0163). By day 15 the difference in sarcomere lengths further increased, with YAP1-KO CMs sarcomeres being on average 0.25 µm shorter (1.66 ± 0.264 µm in WT vs 1.41 ± 0.161 µm in YAP1-KO; multiple comparison *t* test Q = 1%, *P* < 0.0001). At day 30 the difference in sarcomere length narrowed to 0.1 µm but was still significant (1.79 ± 0.169 µm in WT vs 1.69 ± 0.238 µm in YAP1-KO; multiple comparison *t* test Q = 1%, *P* = 0.0004) (Fig. 3E).

Observing that the most dramatic differences in the growth of cell area and sarcomere occurred between days 10 and 15 of differentiation we set at investigating the effect of YAP1 transcriptional and non-transcriptional activity on cardiomyocyte maturation after the onset of beating. First, we inhibited YAP1 transcriptional activity by blocking its nuclear localization by cytoskeletal tension inhibitor latrunculin A, which impairs YAP1 nuclear localization (Fig. 1F). Indeed, sarcomere length was significantly reduced by latrunculin A (WT: 1.797 ± 0.09311 μm vs. latrunculin A: 1.508 ± 0.179 μm; paired *t* test, *P* = 0.046) (Fig. 3F).

Next, we transiently expressed full length YAP1 (YAP-full, able to shuttle to the nucleus) and a YAP1 mutant lacking the c-terminal PDZ domain binding motif FLTWL (YAP-dPDZ, retained in the cytoplasm) [14] in YAP1-KO CMs at D12 (Supplementary Fig. 3, 4A). YAP-full re-expression had observable, but not significant effects on the projected cell area (WT: 2093 ± 397.0 μm^2^, YAP1 KO: 949 ± 220.8 μm^2^, YAP-full: 1381 ± 288.2 μm^2^, YAP-dPDZ: 1917 ± 636.7 μm^2^) after eight days of YAP1 re-expression. Surprisingly, the re-expression of YAP-dPDZ induced slightly higher, although not significant, increase in cell projected area (Fig. 3G).

We next focused on the contractile apparatus of hESC-derived CMs and observed the recovery of sarcomere length after re-expression of both YAP-full and YAP-dPDZ (negative: 1.486 ± 0.073 μm vs YAP-full: 1.687 ± 0.0764 μm, *P* = 0.0305, unpaired *t* test, *N* = 3; negative vs. YAP-dPDZ: 1.714 ± 0.0245, unpaired *t* test, *N* = 2). This effect was significant in both the experimental conditions (Fig. 3H). Intrigued by this observation we assessed the proximity of YAP-Full and YAP-dPDZ to the Z-disc by proximity ligation assay (PLA). Here we observed a positive signal appear when YAP1 and sarcomeric actinin were probed in WT, YAP-Full, and YAP-dPDZ cardiomyocytes (Supplementary Fig. 4B). In addition, we tested cell-growth response of YAP-full and YAP-dPDZ CMs to different concentrations of fibronectin. Again, while not statistically significant, we observed a trend of increased projected cell area in YAP-dPDZ re-expression (Supplementary Fig. 4C).

Taken together, these data indicate that YAP1 presence in the nucleus as induced by ECM remodelling and cytoskeleton assembly, promotes sarcomere assembly in CMs derived from hESCs. They also show that a fraction of the protein is kept closer than 40 nm from the z-disc, as shown by YAP1 proximity with sarcomeric actinin.

### YAP1 modulates CMs electrophysiological properties and intracellular Ca^2+^ dynamics

**Fig. 4:**
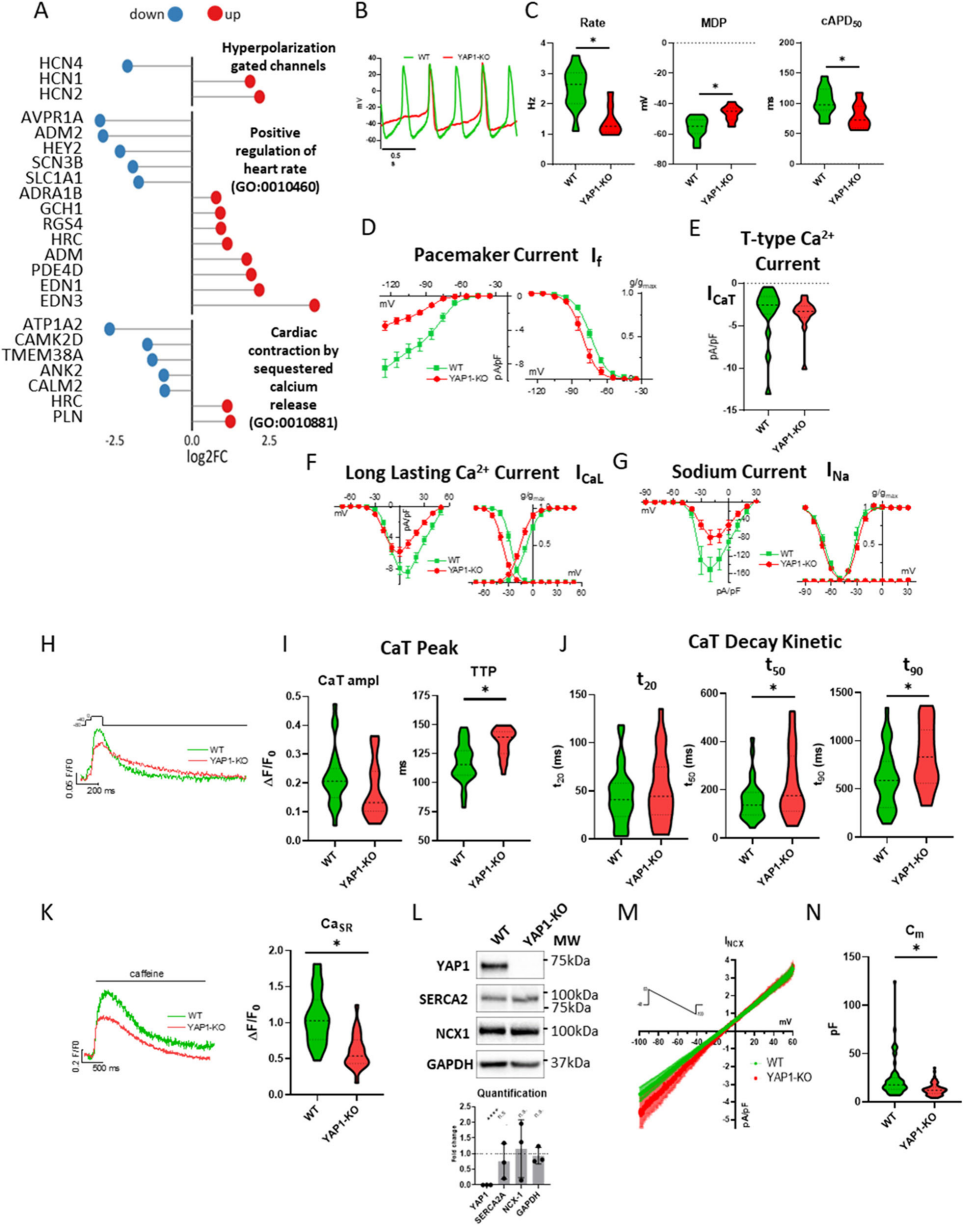
YAP1 regulates maturation of electrophysiological properties and Ca^2+^ dynamic of hESC-CMs. Lollipop plot representation of differential gene expression of indicated gene categories in YAP1-KO compared to WT hESC-CMs (*n* = 3) (**A**). Representative action potentials (AP) (**B**) and quantification of AP parameters in WT (*n* = 12) and YAP1-KO (*n* = 8) CMs: beating rate, maximum diastolic potential (MDP), corrected AP duration at 50% of repolarization (cAPD_50_) (**C**). Pacemaker current (I_f)_ I/V relations (left) and steady-state activation curves (right) in WT (*n* = 23) and YAP1-KO (*n* = 14) CMs (**D**). Quantification of peak T-type Ca^2+^ current (I_CaT_) at −20 mV in WT (*n* = 16) and YAP1-KO (*n* = 16) CMs (**E**). Quantification of long lasting Ca^2+^ current (I_CaL_) I/V relations (left) and steady-state activation/inactivation curves (right) in WT (*n* = 23) and YAP1-KO (*n* = 18) CMs (**F**). Quantification of sodium current (I_Na_) I/V relations (left) and steady-state activation/inactivation curves (right) in WT (*n* = 12) and YAP1-KO (*n* = 18) CMs (**G**). Representative traces of voltage-induced Ca^2+^ transient (CaT) (**H**). Quantification of CaT parameters: time to peak amplitude (left) and (TTP)(right) (**I**). Quantification of CaT decay kinetics at 20%, 50%, 90% decay time in WT (*n* = 29) and YAP1-KO (*n* = 23) CMs (**J**). Representative traces of caffeine-induced transient (CaT) (left) and quantification of the CaT peak amplitude (i.e the SR Ca^2+^ content, Ca_SR_) (right) in WT (*n* = 24) and YAP1-KO (*n* = 22) CMs (**K**). Western blot analysis of SERCA2a and NCX1 protein levels (quantification on bottom) (**L**). Quantification of Mean (±sem) I/V relations of NCX current (I_NCX_) in WT (*n* = 25) and YAP1-KO (*n* = 24) CMs (**M**). Statistics of cell membrane capacitance (C_m_) in WT (*n* = 62) and YAP1-KO (*n* = 66) CMs (**N**). Statistics: unpaired *t* test, *P < 0.05.

The process of CM specification and maturation forces dramatic changes in the ability of CMs to initiate and propagate electrical impulses and generate force [54]. During our RNA-seq experiments, we observed that YAP1 deficiency dysregulated multiple categories of genes modulating CM beating rate and intracellular Ca^2+^ dynamics (Fig. 4A; Supplementary Fig. 5). Specifically, gene expression of Hyperpolarization-activated cyclic nucleotide–gated (HCNs) channel, and key components of Ca^2+^ homeostasis were downregulated (i.e. CALM2, CAMK2D) in YAP1-KO CMs.

To extend our knowledge on the importance of YAP1 for CM maturation from a functional perspective, we analysed the electrophysiological properties and the intracellular Ca^2+^ dynamics of YAP1-KO and WT CMs using patch-clamp and fluorometric techniques, respectively.

At 20 days of differentiation, both YAP1-KO and WT presented spontaneous electrical activity as shown by representative action potential (AP) traces (Fig. 4B). In comparison to WT cells, YAP1-KO CMs beat significantly slower, showed more depolarized maximal diastolic potential (MDP) and shorter AP duration at 50% of repolarization (APD50). To notice, APD50 was corrected to the cell beating rate (cAPD50) to avoid APD changes mainly related to different beating rates (Fig. 4C).

To shed light on the AP rate dysregulation, we measured the pacemaker current (I_f_) current dependent on HCNs expression and well known to contribute to diastolic depolarization rate (DDR). We observed that I_f_ density was significantly reduced in YAP1-KO CMs (Fig. 4D, left). Moreover, the steady-state activation curve was significantly leftward shifted (toward more negative potentials, −7.08 ± 0.17 mV, *P* < 0.05) (Fig. 4D, right), suggesting that both maximal conductance reduction and changes in activation biophysics of If could explain the decreased beating rate of YAP1-KO CMs. Notably, T-type Ca^2+^ current (I_CaT_), potentially contributing to DDR, was not affected by YAP1 deficiency (Fig. 4E).

According to APD_50_ shortening, the Long lasting Ca^2+^ current (I_CaL_) was significantly reduced in YAP1-KO CMs in terms of conductance and biophysics. Indeed, voltage-dependent I_CaL_ availability (Fig. 4F, left) was also affected by YAP1 deficiency; in particular, both steady-state activation and inactivation curves were leftward shifted in YAP1-KO CMs of −7.4 ± 0.2 mV (*P* < 0.05 vs WT) and −9.62 ± 0.17 mV (*P* < 0.05 vs WT) respectively (Fig. 4F, right), suggesting a marked alteration of I_CaL_ voltage dependency in YAP1-KO CMs.

Finally, Na^+^ current density (I_Na_) was markedly reduced in YAP1-KO CMs in comparison to WT (Fig. 4G, left), while its activation/inactivation properties were unaffected by the lack of YAP1 (Fig. 4G, right).

Analogously to electrophysiological properties, Ca^2+^ dynamics also change during CM development and maturation [54]. To study how YAP1 genetical depletion affects Ca^2+^ handling properties in cardiomyocytes, we evaluated YAP1 effects on voltage- and caffeine-induced Ca^2+^ transients (CaT) in voltage-clamped YAP1-KO and WT CMs (Fig. 4H through 4N). We controlled the membrane potential to avoid confounding secondary effects otherwise present in spontaneous beating or field stimulated cells. As can be seen in Fig. 4H, compared to WT CMs, YAP1-KO CMs showed slower voltage-induced CaT onset (quantified as increased CaT time to peak, TTP in Fig. 4I, right) and slower CaT decay, suggesting alterations in EC-coupling machinery and in diastolic Ca^2+^ removal systems. Moreover, CaT amplitude tended to be reduced in YAP1-KO CMs (*p* > 0.05 vs WT) (Fig. 4I, left). In addition, quantification of CaT decay components revealed that the slow component of the CaT decay (quantified as t_50_ and t_90_) significantly increased in YAP1-KO CMs, while the early phase of CaT decay (quantified as t_20_) was unaffected (Fig. 4J). To better estimate the potential effects of YAP1 on sarcoplasmic reticulum (SR) Ca^2+^ content (Ca_SR_), caffeine-induced CaT and the Na^+^/Ca^2+^ exchanger (NCX) current (I_NCX_) activated during caffeine pulse, were quantified at the same time. Both caffeine-induced CaT amplitude (Fig. 4K) and Ca^2+^ influx through NCX (not shown, see Methods) were significantly reduced in YAP1-KO CMs, thus indicating a reduced Ca_SR_ in comparison to WT cells.

Furthermore, western blot evaluation of total NCX1 and SERCA2a protein levels did not highlight changes in YAP1-KO CMs in comparison to WT (Fig. 4L). To further characterize this finding, we also evaluated NCX1 protein expression at the membrane level by measuring I_NCX_ through a dedicated voltage clamp protocol. Consistent with our western blot results, we did not detect any difference in inward and outward conductance of NCX1 in YAP1-KO CMs compared to WT (Fig. 4M). These results are apparently in contrast to the observed slower CaT decay in YAP1-KO CMs and implied an altered distribution of NCX probably related to immature maturation of YAP1-KO CMs, leading to a less efficient EC-coupling.

Electrophysiological quantification of cell dimension through cell membrane capacitance (C_m_) evaluation confirmed the immature/smaller phenotype of YAP1-KO CMs compared to WT (Fig. 4N).

In conclusion, all these measurements indicate that YAP1 reactivation mediated by ECM remodelling might contribute to altered maturation of excitation-contraction coupling mechanisms in cardiomyocytes.

### YAP1 promotes contractile force generation in 3D engineered heart tissues

Our characterization indicated that YAP1 is required for sarcomere assembly and intracellular Ca^2+^ dynamics in hESC-derived cardiomyocytes. Next, we set at investigating whether these YAP1 effects would result in changes in cardiomyocyte contractility. To test the validity of this hypothesis in a relevant 3D model, we generated engineered heart tissues (EHTs) which contract synchronously and spontaneously over a long time.

To increase the relevance of the data obtained in hESC-derived cardiomyocytes, we prepared EHTs from control (WT) and YAP1 deficient (YAP1-KO) human induced pluripotent stem cells (iPSCs) [55] and confirmed impaired maturation in absence of YAP1 (Supplementary Fig. 6). Next, control and YAP1-KO iPSCs differentiated into cardiomyocytes for thirty days, were cast into a collagen hydrogel and followed for 10 days in a culture platform enabling live monitoring of force generation (https://www.optics11life.com/products/cuore/).

**Fig. 5:**
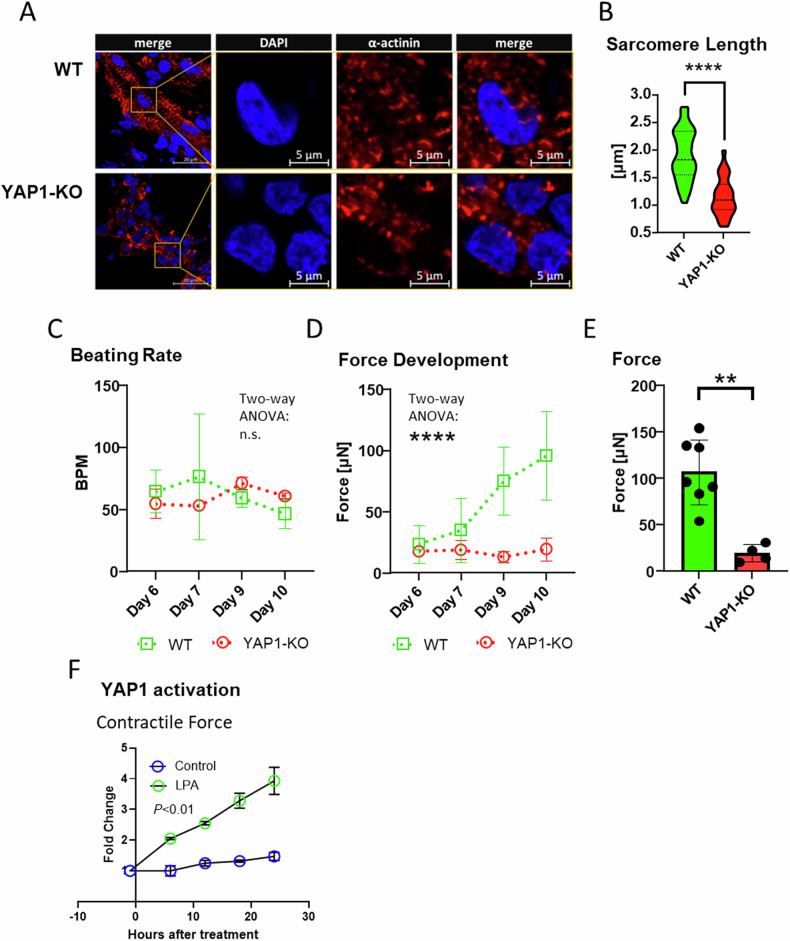
YAP1 promotes contractile force in a model of engineered heart tissues. Cardiomyocytes differentiated from WT and YAP1 deficient induced pluripotent stem cells (iPSCs) for 30 days were cast in 3D microtissues. Longitudinal sections of 3D microtissues stained at day 10 post casting for alpha-actinin (red) counterstained with DAPI (blue) (**A**). Analysis of sarcomere length at day 10 post casting (*P* < 0.0001, unpaired *t* test, *N* = 1, *n* = 63) (**B**). Cuore platform (Optics11 Life) was used to measure beating rate (**C**), development of contraction force at different time points following casting (**D**). Contractile force measurements at day 10 post casting (**E**) (*P* < 0.01, unpaired *t* test, *n*(WT) = 7, *n*(YAP1-KO) = 4). Contraction force of EHTs at day 7 was measured over 24 h period after treatment with YAP activator lysophosphatidic acid (LPA) (all timepoints except −1 h *P* < 0.01, multiple *t* test, *n*(Control) = *n*(LPA) = 3) (**F**). The contraction force values were normalized to pretreatment levels (−1 h). Statistics: *P < 0.05. **P < 0.01.

We first aimed to confirm whether the detrimental effects of YAP1 genetic depletion on sarcomere structure detected in 2D CMs could also be found in 3D complex microtissues like EHTs. We thus stained WT and YAP1-KO EHTs for alpha sarcomeric actinin and found the myofibril content was visibly reduced in EHTs obtained from YAP1 deficient cardiomyocytes (Fig. 5A). As a result, the maturation of the sarcomere in the YAP1 deficient 3D constructs was significantly compromised as measured by sarcomere length (1.918 ± 0.459 μm in WT vs 1.166 ± 0.353 μm in YAP1-KO, unpaired *t* test, *P* < 0.0001) (Fig. 5B).

We next monitored the beat rate and contractile force developed by the EHTs generated from WT and YAP1-KO cells. Initially we did not observe significant differences in the beating rate between control (64.57 ± 17.15 BPM) and YAP1-KO (54.75 ± 11.73 BPM) at day 6 after EHT formation. Over time the beating rate diverged. While control CMs slowed down slightly (46.60 ± 11.61 BPM at day 10), which is consistent with our observations in cardiac organoids [56], the beating rate of YAP1 deficient CMs slightly increased (61 ± 2 BPM) (Fig. 5C). more importantly, significant differences could be detected in the force generation: in fact, while the force produced by control microtissues increased fourfold over time (from 23.73 ± 15.57 μN at day 6 to 95.81 ± 36.32 μN at day 10), the force of YAP1 deficient cardiomyocytes did not change significantly with time in culture (from 17.91 ± 9.863 μN at day 6 to 19.43 ± 9.475 μN at day 10) (Fig. 5D). The comparison of contractile force between control and YAP1 deficient CMs at day 10 - when the power contraction curve in control cells started to plateau – revealed nearly a 5-fold difference in contractile force (Fig. 5E). To further investigate the effect of YAP1 activity on force production in the 3D model of WT cardiomyocytes we used YAP1 activator (lysophosphatidic acid-LPA) for 24 h and we observed a significant increase in the force produced by 20 μM LPA (control vs LPA, multiple *t*-test, *n* = 3) (Fig. 5F).

In conclusion, these data obtained in 3D engineered heart tissues via genetic modification of pharmacological modulation, indicate that YAP1 re-expression in cardiomyocytes, when determined by ECM pathological remodelling, might be pivotal for the development of contractile force.

## Discussion

While targeting mechano-signalling pathways in fibroblasts is becoming a clinical modality [57], the mechanistic understanding of cardiomyocytes cellular response to ECM remodelling has not yet reached this threshold. Together with others, our group demonstrated that the mechanosensitive protein YAP1, a protein which is crucial to foetal cardiomyocyte proliferation [31, 33], is promptly re-expressed in endangered cardiomyocytes following an insult to promote their survival and activate the mutually exclusive hypertrophic or proliferative response [2, 3, 37, 38]. The hypertrophic response was observed in animal models of pressure overload and myocardial infarction in the presence of endogenous YAP1 [5, 58]. The pro-proliferative effects of YAP1 were instead achieved by leveraging the ectopic activation of YAP1. Both the deletion of Hippo effectors (SAV1/2, LATS1/2) or the ectopic expression of YAP1 insulated from Hippo inhibition by mutations at one or multiple serine residues (YAP -S127A, S5A, S8A) can force cardiomyocyte proliferation that promotes regeneration [6, 31, 33, 50]. However, several studies described the downside of constitutively activating YAP1 in the diseased heart. SAV1/2 deletion worsens the response to pressure overload in animal models [36], unrestricted proliferation of CMs leads to heart failure in models in vivo [12, 37]. These observations, together with the observed sarcomere-disrupting effects of YAP1 transcriptional hyperactive mutant S127A described in skeletal muscle [7] together with the deleterious effects of the ectopic expression of YAP1 transcriptional partner TEAD on pressure overloaded heart [35] underscore the need for better understanding of the regulatory mechanisms of hypertrophic/proliferative outcomes of YAP1 reactivation in cardiomyocytes.

Here we set at investigating the role of endogenous YAP1 reactivation in in vitro hESC-CM model by choosing two orthogonal components of ECM pathological remodelling: (i) fibronectin concentration and (ii) increased mechanical strain. To clearly identify YAP1 dependent and independent components of cellular response we used convenient genetic model: two human pluripotent stem cell lines (embryonic and induced pluripotent stem cells) in which YAP1 has been genetically depleted by CRISPR/Cas9.

Initially we found that either the mechanical actuation or fibronectin accumulation are sufficient to trigger YAP1 nuclear shuttling via a mechanosensing pathway which requires the transmission of intracellular tension through F-actin cytoskeleton. These observations are congruent with previous observations in the diseased heart in vivo and other in vitro models [3, 5, 43].

Next, we investigated the broader effects of YAP1 deficiency by using the same CRISPR/Cas9 pluripotent stem cell models. By using bulk RNA-seq and crossing the results with ChIP-seq analysis of the endogenous protein, we assessed the broad landscape of differences in gene expression in YAP1 depleted CMs. YAP1 binding to promoters was associated with increased gene expression confirming the role of endogenous YAP1 as a transcription activator, which is in line with previous published works of endogenous and constitutively active YAP S127A [12, 20]. However, we observed several notable differences with previous reports: we did not observe the shift to more pro-proliferative and foetal phenotype. On the contrary, the largest changes concerned gene ontologies which govern muscle and sarcomere development. In addition to members of Igf and Wnt family [6], system wide regulators of muscle and sarcomere development in mouse, we observed that thin filament, thick filament, and Z-disc proteins (LMOD2, MYH7, MYBPC, CRYAB) [59–63] were regulated by YAP1. The possible interpretation of these discrepancies can lie in the observation that YAP S127A does not only bind to previously identified genomic locations but “reprograms” chromatin to occupy new ones [12]. Similarly, based on the studies using constitutively active YAP1 (or Hippo deletion) we expected reduced proliferation in YAP1-KO hESC [3, 6, 33], but instead observed no differences in single-cell CMs proliferation. Apart from the possible differential activity of the endogenous and constitutively active YAP1, two more factors may influence the proliferative potential of CMs in our in vitro model: sarcomere organization and cell density. Organized sarcomere structures - well established in our D15 CMs - act as a block to CM proliferation (reviewed in [64]). The proliferation rate of hPSC-CMs depends on cell density [65]. Moreover proliferation rate of sparse hPSC-CMs was described to be YAP1 independent in [66].

The main observations contained in our work concern the proliferation independent function of YAP1 on cell growth, myofibril genesis, sarcomere maturation, and force production. YAP1-deficient cardiomyocytes express a lower amount of myosin’s and are not able to form comparable amounts of myofibrils. Furthermore, the few myofibrils that are present in YAP1 depleted cardiomyocytes are not well aligned. Our observations are supported by the smaller cardiomyocytes found in the heart of YAP1 cKO mice [44], proliferation independent effects of YAP1 on myofibril [67] and the pro-hypertrophic effect of endogenous YAP1 in response to injury [5]. Sarcomere maturation – measured as the progressive lengthening of the distance between adjacent z-discs during differentiation – also increases slower in the absence of YAP1. Interestingly, this deficiency can be rescued by re-expression full-length or PDZ binding motif deficient YAP1, which shows compromised nuclear localization and unable to exert any transcriptional activity [14, 68]. While the underlying mechanism of this novel phenomenon remains unexplained, we observed the co-localization of YAP1 at the Z-discs in WT, full-YAP and YAP-dDPZ CMs. YAP1 has been reported to interact with tight junction proteins, cytoskeletal scaffolding complexes [66], and membrane bound complexes to mediate Cdc42 activity in various tissues [22, 23] but the nature of this interaction and its significance in context in cardiomyocytes remains obscure.

Sarcomere development and maturation are interconnected with changes in EC-coupling during heart development. Initially, the combined action of NCX1 and L type Ca^2+^ channel (LTCC, I_CaL_) start oscillatory Ca^2+^ transients, which later develop into regular oscillations, beat frequency increases and its coordination is delegated to pacemaker cells in sinoatrial node (SAN) using mainly funny current (I_f_) flow through HCN channels to set the frequency [69–71]. While YAP1 deficiency causes no observable changes prior to E7.5 (start of detectable heart contractions), at day E9.5 heart rate of YAP1 deficient cardiomyocytes slows down before embryonic lethality occurs between days E10.5-E17 [6]. We found that genes regulating sodium and calcium currents (I_CaL_) connected with maturation were repressed in the absence of YAP1. Consequently, the spontaneous beating rate of YAP1-KO cardiomyocytes was significantly slower, result well explained by HCN4 gene reduced expression, I_f_ density and I_f_ open probability. Of note, Hippo-YAP1 axis has been shown to keep homeostasis in sinoatrial node [72].

Furthermore, YAP1 deficient cardiomyocytes displayed more immature Ca^2+^ handling properties with reduced SR Ca^2+^ content and slower Ca^2+^ transient decay suggesting decreased efficiency of Ca^2+^ removal systems (mainly SERCA2a and NCX1) despite unchanged protein levels of both SERCA2 and NCX1; analogously, the maximal conductance of I_NCX_ was not affected by YAP1 deficiency. Our understanding of the effects of YAP1 transcriptional activity on calcium homeostasis is mixed. While it has been shown that its transcriptional activity through TEAD1 regulates SERCA2a expression and is necessary for adult heart function [73], its overexpression leads to heart failure through the repression of Serca2a expression [35]. In addition, a recent study showed that YAP1 Ca^2+^ transient decay depended on YAP1 activity [44]. Our study supports the case that the activity of endogenous YAP1 is essential for the maturation of Ca^2+^ handling apparatus.

Finally, we assessed the net effect of compromised cardiomyocyte structural and electrophysiological properties on YAP1 deficient cardiomyocytes by measuring cardiomyocyte contractility by generating engineered heart tissues (EHTs) [74, 75].

In this 3D model of heart tissue, we confirmed that YAP1 depletion determines decreased quantity of myofibrils, and reduction in sarcomere length which translates into a reduced ability to produce force. Lastly, we found that YAP1 activator LPA significantly promotes force generation. While LPA has not been observed to increase the inotropy of adult cardiomyocytes [76] its Rho linked activity promotes hypertrophy in neonatal myocytes [76] and promotes actin polymerization [77, 78] which can influence the length of thin filament and thus force generation.

Taken together, our data indicate that YAP1 reactivation both in the nucleus and at the sarcomeres of cardiomyocytes induced by ECM remodelling contributes to myofibril alignment and sarcomere maturation. Our results reveal a novel role for Hippo downstream effector at the sarcomeres in the maturation of cardiomyocyte contractile apparatus and electrophysiological properties.

## Materials and methods

See Supplementary data – Methods

## Supplementary information

Materials and Methods

Supplementary Figures Legends

Supplementary Figure 1: Effect of YAP1 inhibition on sarcomere structure.

Supplementary Figure 2: YAP1 transcriptional regulation of sarcomere associated genes

Supplementary Figure 3: YAP1 re-expression

Supplementary Figure 4: YAP-dPDZ localization and hypertrophic response

Supplementary Figure 5: YAP1 transcriptional regulation of ion channels expression.

Supplementary Figure 6: YAP1 effects on maturation in hIPSC-CMs 2D and 3D

Supplementary Figure 7: Full Western Blots

Supplementary Table 1

Supplementary Table 2

Supplementary Table 3

Supplementary Table 4

Supplementary Table 5

## Data Availability

The datasets generated during and/or analysed during the current study are available from the corresponding author on reasonable request.
