# Supplementary material for: YAP1 reactivation in cardiomyocytes following ECM remodelling contributes to the development of contractile force and sarcomere maturation
Source: Cell Death Discov. 2025 Nov 10;11:518. doi: 10.1038/s41420-025-02793-2 (PMC12603042; doi:10.1038/s41420-025-02793-2)
Supplement: Supplementary file 1 — Materials and Methods [file 41420_2025_2793_MOESM1_ESM.docx]

# Materials and methods

## Cell Culture

### Cell-lines differentiation

The YAP deficient (YAP1‑KO) and isogenicfull H9 (WT or CTRL) human embryonic stem cell lines (hESCs) were a kind gift of Miguel Ramalho-Santos and Han Qin (1). The cells were maintained in an undifferentiated state by culturing them on Matrigel Growth Factor Reduced (1:100 in DMEM/F12, Corning, NY, USA) coated tissue culture plastic plates in complete Essential 8™ Medium (E8, Thermo Fisher Scientific) containing penicillin/streptomycin (0.5%, VWR).

The YAP deficient iPSC cells and the isogenic wild type (hPSCreg ID: UKAi009-A) have been characterized and described in detail before (2). In brief, the iPSCs were derived after informed and written consent using guidelines approved by the Ethic Committee for the Use of Human Subjects at the University of Aachen (permit number: EK128/09). A CRISPR/Cas9 nuclease approach was then used to target exon three of the human YAP1 gene, which is shared in all transcripts. iPSC lines were initially cultured on tissue culture plastic coated with vitronectin (0.5 µg/cm^2^; Stemcell Technologies, Vancouver, Canada) in StemMACS iPS-Brew XF (Miltenyi Biotec GmbH, Bergisch Gladbach, Germany).

Cell differentiation was conducted using previously described protocol (3) with slight modifications by using sequential inhibition of GSK-3 by CHIR99021 (8 µM, Sigma-Aldrich) and WNT by IWP-2 (5 µM, Selleck chemicals) in RPMI 1640 media (Gibco) supplemented with B27 supplement without insulin (RPMI +B27 -Ins) (1x, Gibco), containing penicillin/streptomycin (1%, VWR). Geltrex^TM^ (1:100, Gibco) was added to cell culture media during the first six days of the differentiation. When the cell cultures started contracting the RPMI media was supplemented with B27 containing insulin (RPMI +B27 +Ins) (1x, Gibco). The culture media were exchanged every 2-3 days. Cells on differentiation days 10, 15-20 and 30-40 were used for experiments.

### Single-cell cardiomyocyte culture

At specified timepoints the confluent cell cultures were enzymatically dissociated using Multi Tissue Dissociation Kit 3 (Miltenyi Biotec) and replated at 10.000 cells/cm^2^ in RPMI +B27 +Ins media with ROCK inhibitor Y27632 (5 µM, Selleck chemicals) supplemented RPMI media to facilitate cell attachment. The media was changed the next day for RPMI +B27 + Ins media without ROCK Inhibitor. The experiments on single cell cultures were performed on day four after replating. The fourth day timepoint was selected as a day when the replated single cell cardiomyocytes resumed spontaneous contraction.

### Surface coating

Single cardiomyocyte cell culture dishes were coated with Matrigel Growth Factor Reduced (1:20 in RPMI, Gibco, Corning). For ECM concentration experiments and mechanical actuation experiments on Mechanoculture FX2 plates fibronectin coating (1 µg/ml and 10 µg/ml, in PBS StemCell Technologies) at 37°C for 1 hour were used.

### Cell treatments

For actin tension inhibition experiments, single cardiomyocytes were treated with latrunculin A (250 nM, Cayman Chemical), verteporfin(0.05 μM, Selleckchem), 1-Oleoyl Lysophosphatidic Acid (LPA) (20 µM) for 24 hours.

### PDMS-preparation

Mixtures of Sylgard 184 and Sylgard 527 were used to prepare compliant materials with Young modulus 10 kPa and test stiffness as published previously (4,5).

### Mechanical actuation protocol

The mechanical stimulation experiments were conducted on single cell cardiomyocyte cultures seeded on compliant stretching plates supplied with Mechanoculture FX2 device (CellScale). Confluent cultures of beating cardiomyocytes at day 15-20 of differentiation were enzymatically dissociated and seeded at 10.000 cells/cm^2^ on the fibronectin coated stretching plates. After four days of single cell culture, the cell substrate was stretched to 120% of the well length during one second and kept static for next 24 hours, before fixation with 4% PFA followed by immunofluorescence staining.

### Engineered Heart Tissues (EHT) and measurements of contractile force

EHTs were generated using Cuore device (Optics11-Life, Amsterdam, NL), a pillar-based system for standard 24-well plates that uses integrated fibre optical sensing and Electrical Pulse Stimulation (EPS) for continuous electrical stimulation and real-time recording of contractile activity (6). The 24-well casting plate was pretreated O/N with 1% Pluronic acid (Sigma) and kept at 4 °C for 24 hours to prevent cell attachment. The day after, 0.5 × 10^6^ WT and YAP1-KO hiPSC-CMs at day 30 of differentiation were mixed, on ice, with Collagen type I (0,072 mg/EHTs, TeloCol®-6, Advanced Biomatrix), 0,003 N NaOH and RPMI +B27 + Ins supplemented with 20% FBS and 5 µM Y27632. The cell-hydrogel mixture was distributed into 24-well casting plate and placed inside the Cuore device equipped with an optical fibers plate containing an array of 24 couples of cantilevers. The device was then placed inside the incubator in order to allow the condensation of the cell/hydrogel mixture for 1 hour and then more media were added. Twenty-four hours later, the media were substituted with RPMI +B27 + Ins. Three/four days from the casting, the array of cantilevers with the attached EHTs was transferred into a new 24-well plate (Corning™ Costar™). Half of the medium in each well media was replaced every 48h hours. were kept in culture for 10 days. The estimated contractile force generated from WT and YAP-KO EHTs was recorded at different time points from day 6 to day 10. The data were analysed using an integrated computer software to calculate the absolute contractile force and the beating.

### Viral particles production

Plasmid containing full length YAP1 with T2A-mCherry tag (#74942, Addgene) or PDZ binding domain deficient YAP1 (#59147/Addgene) was transfected together with envelope expressing plasmid 9 - PMS2.G (#12259, Addgene), and empty backbone packaging plasmid PSPAX2 (#12260, Addgene) into HEK293T cells using FuGENE® HD Transfection Reagent according to manufacturer’s instructions. The media with HEK293T produced viral particles was collected daily for 4-5 days post transfection and concentrated using Vivaspin20 100kDa protein concentrators to achieve 30x concentrated stock of viral particles. The YAP1 deficient cardiomyocytes were transduced using 8x concentrated viral particles in presence of Polybrene Transfection Reagent (7,5 µg/ml, MERCK). After visual confirmation of YAP1 expression by means of mCherry at day four post transduction, cardiomyocytes were replated into the single cardiomyocyte cell cultures for experiments.

## Immunofluorescence

### Adherent cultures

Immunofluorescence staining was executed as published previously (7). In detail, cells were fixed by 4% PFA for 10 minutes, washed and stored in PBS prior to staining. Permeabilization and blocking of non-specific binding epitopes were done using 0.1% and 0.05% Triton X-100 and Tween-20 respectively in 1% BSA in PBS. Incubation with primary antibodies at 4°C overnight in 0.05% Tween-20 in 1% BSA in PBS was followed by washes in 0.05% Tween-20 PBS before incubation with secondary antibodies labelled with fluorescence labels. For primary and secondary antibodies see Supplementary Table 4.

### Engineered heart tissues

The samples were fixed in 4% PFA for 1 h at room temperature (RT) and then incubated with 15% sucrose solution (Sigma Aldrich) supplemented with 0.03% eosin (Sigma Aldrich) overnight at 4°C. EHTs were then embedded in OCT solution (Leica), frozen in cassettes embedded in isopentane (VWR) cooled with dry ice and stored at -80℃ until cryosectioning. The frozen EHTs were cryosectioned using the CryoStar NX70 Cryostat (ThermoFisher Scientific). [Frozen sections](https://www.sciencedirect.com/topics/biochemistry-genetics-and-molecular-biology/frozen-section) were cut at 10 μm thickness, placed onto Menzel Gläser, SuperFrost® Plus slides (ThermoFisher Scientific) and stored at -20℃ until their immunostaining following the same protocol as described above. The slides were mounted with Fluoromount-G (Invitrogen). For primary and secondary antibodies see Supplementary Table 4.

### EdU proliferation assay

Single cell cultures of cardiomyocyte were exposed to base analogue EdU (10 µM, BaseClick) in cell culture medium for thirty minutes. Following the exposure, cell cultures were washed with PBS and fixed for immunofluorescence staining. The EdU visualization preceding immunofluorescence staining was performed according to manufacturer instructions.

**Ligation Proximity Assay**

Ligation Proximity Assay was purchased from Sigma Aldrich and used according to manufacturer instructions using a rabbit developed YAP1 antibody (#4912 CST) and mouse developed anti Sarcomeric Actinin (A7811 Sigma Aldrich).

## Western-Blotting

Confluent cell cultures of spontaneously beating cardiomyocytes were enzymatically dissociated using Tryple enzyme and collected in cold PBS. The pellets were snap frozen and stored at -80°C. The pellets were resuspended in 1% SDS lysis buffer as described previously(8). Protein concentration was measured using Pierce™ BCA Protein Assay Kit (Thermofisher Scientific) and normalized to 1 mg/ml. Laemli was added to the samples and they were denaturated at 95°C for five minutes. Twenty micrograms of protein per sample were loaded into each lane of 4-20% Mini-PROTEAN® TGX™ Precast Protein Gels (Bio-Rad). The proteins were transferred to nitrocellulose membrane using semidry Trans-Blot® Turbo™ Transfer system (Bio-Rad) and immunodetected using specific primary antibodies diluted in 5% BSA or 5% low fat milk overnight. The signal of secondary HRP conjugated antibodies was visualized by Clarity Western ECL Substrate (Bio-Rad) and acquired using ChemiDoc XRS+ system (Bio-Rad). Integrated density of the signal of detected protein was quantified using Image Lab Software (Bio-Rad). See Supplementary Table 4 for list of used antibodies and Supplementary Figure 7 for full Western Blots.

## Image acquisition

The confocal microscope ZEISS LSM780 (Zeiss, Oberkochen, Germany) was used to acquire images using 10x magnification for tile scans, 40x for cell size measurement and YAP1 localization, and 63X magnification for sarcomere length analysis according to manufacturer’s description. ZEISS Elyra 7 with lattice SIM module was used to acquire images with sub-0.1 µm resolution in all three dimensions. The SIM processing was done using manufacturer algorithm.

## Image analysis

### Cell area and YAP1 localization

Cell area and YAP1 localization was measured using a combined pipeline of Ilastik (9) and CellProfiler 4 (10) custom pipeline. The cardiac troponin channel was used to segment cardiomyocytes (cell object), DAPI channel to segment nuclei. The nuclei objects were subtracted from the cell objects to create a cytoplasm object. For all objects area, circularity, and intensity of immunofluorescence of YAP1 signal were measured and used for further analysis. On average 40-100 cells were measured for every experimental condition per biological replicate. Three independent batches of cardiomyocyte differentiation were used for each experiment.

### Sarcomere length

Sarcomere length was measured using combination of Fiji (11) and custom R application on 63x objective acquired images. First intensity line profiles along one to four myofibril segments containing ≥ 3 Z-discs per cell were selected. The intensity values and region of interest snapshots were saved in csv and tif format respectively for future validation. Number and spacing of peaks were measured using R based application (available at <https://vinarsky.shinyapps.io/sarcomere/>). The quality of myofibril segments and Z-disc detection was independently validated by another group member. Fifteen to thirty cells were measured per condition in each biological replicate. Three independent batches of cardiomyocyte differentiation were used for each experiment. In YAP1 re‑expression experiments the nuclear intensity of YAP1 was used to identify YAP1 rescued cardiomyocytes.

### Differentiation efficiency

Differentiation efficiency was measured in single cell cultures of cardiomyocytes. Twenty‑seven independent view fields of DAPI and cardiomyocyte markers (cardiac troponin/sarcomeric actinin) were acquired using confocal microscope at 10x objective per experimental condition. After pre-processing in Ilastik pipeline, percecustom Cell Profiler 4 pipeline DAPI channel was used to segment nuclei, cardiomyocyte marker channel was used to identify regions covered by cardiomyocytes. The percentage of cardiomyocytes was calculated as a ratio of nuclei found in cardiomyocytes covered area to total number of nuclei. Three (WT) and two (YAP1-KO) independent batches of differentiation were used for each experiment.

### RNA isolation, reverse transcription PCR and Real-Time PCR of EHTs

For RNA isolation, iPSC-CMs at day 40 of differentiation were dissociated with MACS Multi Tissue Dissociation Kit 3 (Miltenyi Biotec) according to manufacturer’s instructions. WT and YAP1-KO EHTs were snap frozen in liquid nitrogen and stored at -80 °C before thawing them O/N at -20 °C in RNA LATER-ICE (ThermoFisher). RNA was extracted using High Pure RNA Isolation Kit (Roche) according to manufacturer’s instructions and quantified with Nanodrop 2000 Spectrophotometer (Thermo Fisher Scientific).

RNA reverse transcription was performed with the Transcriptor First Strand cDNA Synthesis Kit (Roche) according to manufacturer’s instructions. One µg of total RNA was used for each sample. RT-PCR was performed using StepOnePlus System (ThermoFisher) with qPCRBIO SyGreen® Mix Hi-ROX (PCR Biosystems) with respective primers (Supplementary Table 5).

## ChIP-seq

Spontaneously contracting confluent cultures of iPSCs derived cardiomyocytes were differentiated in 60 mm tissue culture plates (one plate per sample) until day sixteen of differentiation. Chromatin was immunoprecipitated from three technical replicates using a ChIPgrade anti-YAP antibody (CST14074, Cell Signaling Technologies) and following the manufacturer’s protocol (Pierce™ Agarose ChIP Kit, Thermo Fisher Scientific). A control ChIP was performed using rabbit immunoglobulins (IgG). The samples were eluted in 30 μL eluting buffer and stored at -80 °C before analysis.

For library preparation, the size distribution of each ChIP DNA sample was assessed by running a 1 µl aliquot on Agilent High Sensitivity DNA chip using an Agilent Technologies 2100 Bioanalyzer (Agilent Technologies, Santa Clara, CA, USA). The concentration of each DNA sample was determined using a high sensitivity Quant-iT™ dsDNA Assay Kit and a Qubit Fluorometer (Thermo Fisher Scientific). Purified ChIP DNA (10 µg) was used as the starting material for sequencing libraries preparation. Indexed libraries were prepared using a TruSeq ChIP Sample Prep Kit (Illumina Inc., San Diego, CA, USA). The libraries were sequenced (single read, 1x50 cycles) at a concentration of 10 pm/lane on a HiSeq 2500 (Illumina Inc.).

Data analysis was performed by Genomix4Life S.r.l. (Salerno, Italy). The raw sequence files generated (.fastq) underwent quality control analysis using FastQC (http://www.bioinformatics.babraham.ac.uk). The reads were aligned to the human genome (assembly hg19) using bowtie (12), allowing up to one mismatch and considering uniquely mappable reads. The reads of replicates and corresponding input samples were merged for peaks calling as previously described(13). ChIP-Seq peaks were identified and analysed using HOMER Motif Database (-F: 2.0, -L: 2.0 and -C: 1.0) with a false discovery rate < 0.01 (14). The assignment of YAP peaks to target genes was obtained using the web tool ChIPSeek (15).

Through this step, it was possible to assign peaks to the transcription start site (by default defined from - 1 kb to + 100 bp), transcription termination site (by default defined from -100 bp to + 1 kb), Exon (Coding), 5’-untranslated region (UTR) Exon, 3’ UTR Exon, Intronic or Intergenic regions. As some annotations overlap, the following order of priority was chosen for the assignment:

- Transcription start site (by default defined from - 1 kb to + 100 bp)
- Transcription termination site (by default defined from -100 bp to + 1 kb)
- CDS exons
- 5’ UTR exons
- 3’ UTR exons
- **CpG islands
- **Repeats
- Introns
- Intergenic

## The peaks were annotated through GeneHancer (16) to connect the peaks in enhancers and promotores to specific genes. The list of enhancers and genes and the method of annotation is described a Supplementary Table 3.

## RNA-seq and data analysis

Spontaneously contracting confluent cultures of control and YAP1 deficient hESCs derived cardiomyocytes were differentiated in 60 mm tissue culture plates (one plate per sample) until day sixteen of differentiation.

Library was prepared using NEBNext® Ultra™ II Directional RNA Library Prep Kit for Illumina® with NEBNext® Poly(A) mRNA Magnetic Isolation Module and NEBNext® Multiplex Oligos for Illumina® (Dual Index Primers Set 1). Kits were employed according to manufacturers’ protocol, input for library preparation was 200-300 ng total RNA.

Sequencing was done on Illumina NextSeq 500 using NextSeq 500/550 High Output v2 kit (75 cycles). We have done single-end 75bp sequencing in multiple sequencing runs until all samples had at least 30 million passing filter reads. Fastq files were generated using bcl2fastq software without any trimming.

The quality of the raw sequencing data was assessed using FastQC (https://www.bioinformatics.babraham.ac.uk/projects/fastqc/) and aligned to the hg38 reference genome using the TopHat2 aligner (17). Raw gene counts were obtained by calculating reads mapping to exons and summarized by genes using reference gene annotation (Ensembl 90; Homo sapiens GRCh38.p10, GTF) by HTSeq (18).

Differential gene expression was performed using DESeq2 bioconductor package. Genes were considered as differentially expressed when the Benjamini-Hochberg adjusted *P*value ≤ 0.05 and log2 fold-change (log2FC) ≥ 1.5.

The Gene Ontology (GO) categories were downloaded from AmiGO 2 repository (<https://amigo.geneontology.org>).

## Electrophysiology

After 48 hours, dissociated CMs were recorded at physiological temperature (36 ± 1°C) using the patch-clamp technique in I-clamp or V-clamp mode in whole-cell configuration. The amplifier Axopatch 200B, the Digidata 1550, and the pClamp 10.0 software (Molecular Devices, LLC) were used for data collecting. 10 kHz sampling and 1–5 kHz filtering were applied to the data. Clampfit 10.0 (Molecular Devices, LLC) in conjunction with Origin Pro 9 (OriginLab) was used to analyse the data.

Spontaneous APs were recorded in CMs superfused with Tyrode solution (pH 7.4) containing (mM): 137 NaCl, 5 KCl, 2 CaCl_2_, 1 MgCl_2_, 10 D-glucose, 10 Hepes–NaOH. Patch pipettes were filled with the intracellular-like solution containing (mM): 120 KCl, 20 Na–HEPES, 10 MgATP, 0.1 EGTA–KOH, and 2 MgCl_2_ (pH 7.1). The following AP parameters were analyzed: rate (Hz), maximum diastolic potential (MDP, mV), AP duration at 50% of repolarization (APD50).

To record the funny current (I_f_), the extracellular solution contained (mM) 110 NaCl, 0.5 MgCl_2_, 1.8 CaCl_2_, 5 Hepes-NaOH, 30 KCl, supplemented with 1 BaCl_2_ and 2 MnCl_2_. I_f_ was activated from a holding potential (hp) of -30 mV applying 10 mV hyperpolarizing voltage steps to the range of -35/-125 mV long enough to reach steady-state of activation, followed by a fully activating step at -125 mV.

T-type Ca^2+^ current (ICaT) was isolated in Na^+^-free (replaced with equimolar TEA Cl) Tyrode solution, containing 5 mM CaCl_2_ and supplemented with nifedipine (0.01 mM), TTX (0.01 mM) and 4-aminopyridine (2 mM) to block L-type Ca^2+^ channels, Na^+^ channels and K^+^ channels respectively; K^+^ ions in the pipette solution were replaced by equimolar CsCl and TEACl. ICaT was isolated as Ni^2+^ (0.1 mM)-sensitive current, applying depolarizing voltage steps to -20 mV (hp of -80 mV).

L-type Ca^2+^ current (I_CaL_) was recorded superfusing the cells with (mM) 135 NaCl, 10 CsCl, 1 CaCl^2^, 1 MgCl^2^, 5 HEPES, 10 glucose, supplemented with 0.01 tetrodotoxin (TTX). The intracellular solution contained (mM): 135 CsCl, 1 MgCl^2^, 4 ATP (sodium salt), 0,1 GTP (sodium salt), 5 EGTA–KOH, 5 HEPES-KOH (pH 7.2). I_CaL_ was recorded as nifedipine (0.01 mM)-sensitive current, applying depolarizing voltage steps, 10 mV each, to the range of -40/ +50 mV, followed by a step at 0 mV (hp of -50 mV).

To dissect Na^+^ current (I_Na_), Tyrode solution was supplemented with 0,01 mM nifedipine. I_Na_ was recorded as TTX (0.03 mM)-sensitive current, applying depolarizing voltage steps, 10 mV each, to the range of -80/ +60 mV, followed by a step at -20 mV (hp of -90 mV).

The I_NCX_ was evaluated as the Ni^2+^ (10 mM)-sensitive current during voltage ramps (100 mV/s) from +60 mV to −100 mV (hp −40 mV). The external solution contained (mM): 135 NaCl, 10 CsCl, 1 CaCl_2_, 1 MgCl_2_, 10 Hepes-NaOH, 10 TEA-Cl, 10 D-glucose (pH 7.35) to which 0.2 mM BaCl_2_, 0.005 mM nifedipine, 0.05 mM lidocaine, and 1 mM ouabain were added to block K^+^, Ca^2+^, Na^+^ channels, and the Na^+^/K^+^ pump, respectively. Pipette was filled with (mM): 140 CsOH, 75 aspartic acid, 20 NaCl, 10 CaCl^2^, 10 HEPES, 20 EGTA, 20 TEA-Cl, 5 MgATP (pH 7.3). I_NCX_ density at −80 mV and + 40 mV was taken as representative of the forward and the reverse mode working direction of NCX, respectively.

Current densities were calculated by measuring in each cell the membrane capacitance (Cm). The steady-state activation and inactivation curves were obtained from normalized tail currents or conductances and interpolated with the Boltzmann equation.

The analyses were carried out in parallel between WT and YAP1-KO derived hESC-CMs (N ≥ 3). Significant alterations have been delineated between the two groups by *P* < 0.05 with Student’s *t* test comparison after checking for normal distribution of data with the Shapiro–Wilk test.

## Intracellular Ca^2+^ dynamics

Cytosolic Ca2+ was recorded in single V-clamped CMs loaded with Fluo4-AM (10 µM) at physiological temperature as previously described (19). Cells were incubated for 30 min with Fluo4 at room temperature and then the dye was washed for at least 10 min to allow its de-esterification. Fluo4 was excited at 488 nm, and the emission was collected through a 530 nm band-pass filter, converted to voltage, low-pass filtered (200 Hz) and digitized at 2 kHz after further low-pass digital filtering (FFT, 50 Hz).

Cells were superfused at physiological temperature with the Tyrode’s solution (mM) containing: 154 NaCl, 4 KCl, 2 CaCl_2_, 1 MgCl_2_, 5 HEPES/NaOH, and 5.5 D-glucose (pH 7.35) to which 1 mM BaCl_2_ and 2 mM 4-aminopyridine were added to block K^+^ channels. Patch-clamp pipettes were filled with (mM) 23 KCl, 110 KAsp, 0.04 CaCl_2_, 3 MgCl_2_, 5 HEPES-KOH, 0.1 EGTA-KOH, 0.4 NaGTP, 5 Na_2_ATP, 5 Na_2_-phosphocreatine and 0.01 mM Fluo4-K^+^ salt (pH 7.3). Ca^2+^ transients (CaT) were evocated at 0.5 Hz during 100 ms steps to 0 mV after 50 ms step to -35 mV to inactivate Na^+^ channels. Sarcoplasmic reticulum (SR) Ca^2+^ content (Ca_SR_) was estimated at steady state by measuring CaT amplitude elicited by electronically timed (10 mM) caffeine pulse after 10s at -80 mV. Fluo4 signal was normalized to the resting fluorescence (F0) before caffeine superfusion.

## Statistical analysis

All data are expressed as means± SD unless otherwise specified. The statistical analyses were performed by using GraphPadPrism 8.0 software or R. The null hypothesis was rejected when *P*<0.05. Sample sizes were not pre-determined but were chosen based on previous publications(20,21). To improve the reproducibility of the study and avoid pseudo-replication, mean values of separate biological replicates (independent batches of cardiomyocyte differentiation) were used to calculate the final statistics. Statistical outliers were removed both at the level of individual measurements inside each biological replicate and means of biological experiments using GraphPadPrism 8.0 inbuilt ROUT method with Q = 1%. Number of biological replicates (N) and number of measurements (n), and the statistical tests used are indicated in figure legends and in data files. In case of cell morphometry “n” corresponds to number of individual cells. For morphometric analysis, paired t-test (ptt) was used for experiments with a mechanical actuation and latrunculin A treatment, otherwise unpaired t-test with Welch correction (utt) was used. Electrophysiological data and Ca^2+^ dynamics are shown as mean ± SEM

# References

1. Qin H, Hejna M, Liu Y, Percharde M, Wossidlo M, Blouin L, et al. YAP Induces Human Naive Pluripotency. Cell Rep. 2016 Mar;14(10):2301–12.

2. Zeevaert K, Goetzke R, Elsafi Mabrouk MH, Schmidt M, Maaßen C, Henneke AC, et al. YAP1 is essential for self-organized differentiation of pluripotent stem cells. Biomater Adv. 2023 Mar 1;146:213308.

3. Lian X, Zhang J, Azarin SM, Zhu K, Hazeltine LB, Bao X, et al. Directed cardiomyocyte differentiation from human pluripotent stem cells by modulating Wnt/β-catenin signaling under fully defined conditions. Nat Protoc. 2013 Jan;8(1):162–75.

4. Palchesko RN, Zhang L, Sun Y, Feinberg AW. Development of Polydimethylsiloxane Substrates with Tunable Elastic Modulus to Study Cell Mechanobiology in Muscle and Nerve. PLOS ONE. 2012 Dec 11;7(12):e51499.

5. Vinarský V, Martino F, Forte G, Šleichrt J, Rada V, Kytýř D. DEFORMATION RESPONSE OF POLYDIMETHYLSILOXANE SUBSTRATES SUBJECTED TO UNIAXIAL QUASI-STATIC LOADING. Acta Polytech CTU Proc. 2019 Dec 6;25:79–82.

6. Iuliano A, Haalstra M, Raghuraman R, Bielawski K, Bholasing AP, van der Wal E, et al. Real-time and Multichannel Measurement of Contractility of hiPSC-Derived 3D Skeletal Muscle using Fiber Optics-Based Sensing. Adv Mater Technol. 2023;8(22):2300845.

7. Pagliari S, Vinarsky V, Martino F, Perestrelo AR, Oliver De La Cruz J, Caluori G, et al. YAP–TEAD1 control of cytoskeleton dynamics and intracellular tension guides human pluripotent stem cell mesoderm specification. Cell Death Differ. 2021 Apr;28(4):1193–207.

8. Vinarsky V, Krivanek J, Rankel L, Nahacka Z, Barta T, Jaros J, et al. Human embryonic and induced pluripotent stem cells express TRAIL receptors and can be sensitized to TRAIL-induced apoptosis. Stem Cells Dev. 2013 Jun 27;

9. Berg S, Kutra D, Kroeger T, Straehle CN, Kausler BX, Haubold C, et al. ilastik: interactive machine learning for (bio)image analysis. Nat Methods. 2019 Dec;16(12):1226–32.

10. Stirling DR, Swain-Bowden MJ, Lucas AM, Carpenter AE, Cimini BA, Goodman A. CellProfiler 4: improvements in speed, utility and usability. BMC Bioinformatics. 2021 Dec;22(1):433.

11. Schindelin J, Arganda-Carreras I, Frise E, Kaynig V, Longair M, Pietzsch T, et al. Fiji: an open-source platform for biological-image analysis. Nat Methods. 2012 Jul;9(7):676–82.

12. Langmead B, Trapnell C, Pop M, Salzberg SL. Ultrafast and memory-efficient alignment of short DNA sequences to the human genome. Genome Biol. 2009;10(3):R25–R25.

13. Uusküla-Reimand L, Hou H, Samavarchi-Tehrani P, Rudan MV, Liang M, Medina-Rivera A, et al. Topoisomerase II beta interacts with cohesin and CTCF at topological domain borders. Genome Biol. 2016 Aug;17(1):182–182.

14. Heinz S, Benner C, Spann N, Bertolino E, Lin YC, Laslo P, et al. Simple Combinations of Lineage-Determining Transcription Factors Prime cis-Regulatory Elements Required for Macrophage and B Cell Identities. Mol Cell. 2010 May;38(4):576–89.

15. Chen TW, Li HP, Lee CC, Gan RC, Huang PJ, Wu TH, et al. ChIPseek, a web-based analysis tool for ChIP data. BMC Genomics. 2014 Jun;15(1):539–539.

16. Fishilevich S, Nudel R, Rappaport N, Hadar R, Plaschkes I, Iny Stein T, et al. GeneHancer: genome-wide integration of enhancers and target genes in GeneCards. Database J Biol Databases Curation. 2017 Jan 1;2017:bax028.

17. Kim D, Pertea G, Trapnell C, Pimentel H, Kelley R, Salzberg SL. TopHat2: accurate alignment of transcriptomes in the presence of insertions, deletions and gene fusions. Genome Biol. 2013;14(4):R36.

18. Anders S, Pyl PT, Huber W. HTSeq—a Python framework to work with high-throughput sequencing data. Bioinformatics. 2015 Jan 15;31(2):166–9.

19. Benzoni P, Arici M, Giannetti F, Cospito A, Prevostini R, Volani C, et al. Striatin knock out induces a gain of function of INa and impaired Ca2+ handling in mESC-derived cardiomyocytes. Acta Physiol Oxf Engl. 2024 May 15;e14160.

20. Al Sayed ZR, Jouve C, Seguret M, Ruiz-Velasco A, Pereira C, Trégouët DA, et al. Rod-shaped micropatterning enhances the electrophysiological maturation of cardiomyocytes derived from human induced pluripotent stem cells. Stem Cell Rep. 2024 Oct;19(10):1417–31.

21. Monroe TO, Hill MC, Morikawa Y, Leach JP, Heallen T, Cao S, et al. YAP Partially Reprograms Chromatin Accessibility to Directly Induce Adult Cardiogenesis In Vivo. Dev Cell. 2019 Mar 25;48(6):765-779.e7.
