# Supplementary material for: YAP1 reactivation in cardiomyocytes following ECM remodelling contributes to the development of contractile force and sarcomere maturation
Source: Cell Death Discov. 2025 Nov 10;11:518. doi: 10.1038/s41420-025-02793-2 (PMC12603042; doi:10.1038/s41420-025-02793-2)
Supplement: Supplementary file 2 — Supplementary Figures Legends [file 41420_2025_2793_MOESM2_ESM.docx]

**Supplementary Figures Legends**

**Supplementary Figure 1: Effect of YAP1 inhibition on sarcomere structure.** Representative images of WT hESC-CMs cardiomyocytes untreated (Control) and latrunculin A treated (250 nM, 24 hours) (Latrunculin A) (A) or YAP inhibitor Verteporfin (B, left). Quantification of projected cell area (B, right).

**Supplementary Figure 2: YAP1 transcriptional regulation of sarcomere associated genes.** Lollipop graph representation of up to 15 most differentially expressed (|log2FC|) genes associated with sarcomere structures in YAP1-KO hESC-CMs.

**Supplementary Figure 3: YAP1 re-expression.** Western blot quantification of WT and YAP deficient cardiomyocytes (YAP-KO) mock transfected or transduced with full length YAP1 (YAP-full) or PDZ binding domain motif deficient YAP1 (YAP-dPDZ) (A). Representative images of YAP1 localization in WT, YAP-KO, reexpressed YAP1-full, and reexpressed YAP-dPDZ in YAP deficient cardiomyocytes (B).

**Supplementary Figure 4: YAP-dPDZ localization and hypertrophic response.** Quantification of WT and YAP-dPDZ localization in YAP-KO cardiomyocytes (p=0.0001, unpaired t test with Welsh’s correction, n(WT)=17 n(dPDZ)=37 )(A). Representative images of proximity ligation assay (PLA) between YAP1 and alpha Sarcomeric Actinin in WT, and YAP-KO cardiomyocytes transduced with full length YAP1 or PDZ binding domain motif deficient YAP1 (B). Quantification of cell area of WT, YAP-KO, full-YAP1, and YAP-dPDZ seeded on different concentrations of fibronectin coated TCPS (C).

**Supplementary Figure 5: YAP1 transcriptional regulation of ion channels expression.** Lollipop graph representation of up to 15 most differentially expressed (|log2FC|) ion channels in YAP1-KO hESC-CMs.

**Supplementary Figure 6: YAP1 effects on maturation in hIPSC-CMs 2D and 3D.** Cardiomyocytes differentiated from WT and YAP1 deficient induced pluripotent stem cells (iPSCs) for 30 days were cast in 3D microtissues or kept in 2D adherent conditions for further ten days. Maturation associated changes in gene expression visualized as ratios left to right: myosin heavy chains isoforms (MYH7/MYH6), myosin light chains (MYL2/MYL7), and cardiac troponin I isoforms (TNNI3/TNNI1) expression in WT and YAP1-KO hIPSC-CMs at day 40 of differentiation in 2D and 3D cultures was quantified by RT-PCR (N=1, n=2).

**Supplementary Figure 7: Full Western Blots.**

After electrophoretic transfer of protein from gels to nitrocellulose membranes, the membranes were cut into parts according to molecular weights of interest and incubated with specific antibodies. The signal specific for selected protein (HRP visualized by ECL) and colorimetric image of the membrane were acquired in two separate channels using ChemiDoc MP (Bio-Rad, CA, USA) and merged in Image Lab^TM^ 6.0.1 software (Bio-Rad, CA, USA) to determine molecular weights. The signal was measured using Quantity Tools in Image Lab^TM^ 6.0.1 software (Bio-Rad, CA, USA) on the image of HRP signal. Upper part (>150kDa) of Gel1 was used to detect betaMHC (A top), and after stripping was used to detect panMHC (A bottom). Gel 1 middle part (50kDa-100kDa) was used to detect NCX1 (B top), and after stripping and reincubation YAP1(B bottom). Upper part (50kDa-250kDa) of Gel2 was used to detect SERCA2 (C). Lower part (37kDa-25kDa) of Gel2 was used to detect GAPDH (D top). To increase visibility of weight markers on membrane, the membrane only image with increased contrast was used (D middle). Lower part of membrane was then stripped and used to detect MLC2A (D bottom). Three independent biological replicates of YAP1 re-expression, GAPDH was cut from the same membrane (E) Molecular weights were determined using Precision Plus Protein Dual Color Standard (Bio-Rad, CA, USA). Non specific bands are indicated by red asterix placed below the band. The first two lanes were removed from analysis after failing internal quality control of samples and were not used in analysis.
