# Supplementary figures and images for: YAP1 reactivation in cardiomyocytes following ECM remodelling contributes to the development of contractile force and sarcomere maturation
Source: Cell Death Discov. 2025 Nov 10;11:518. doi: 10.1038/s41420-025-02793-2 (PMC12603042; doi:10.1038/s41420-025-02793-2)

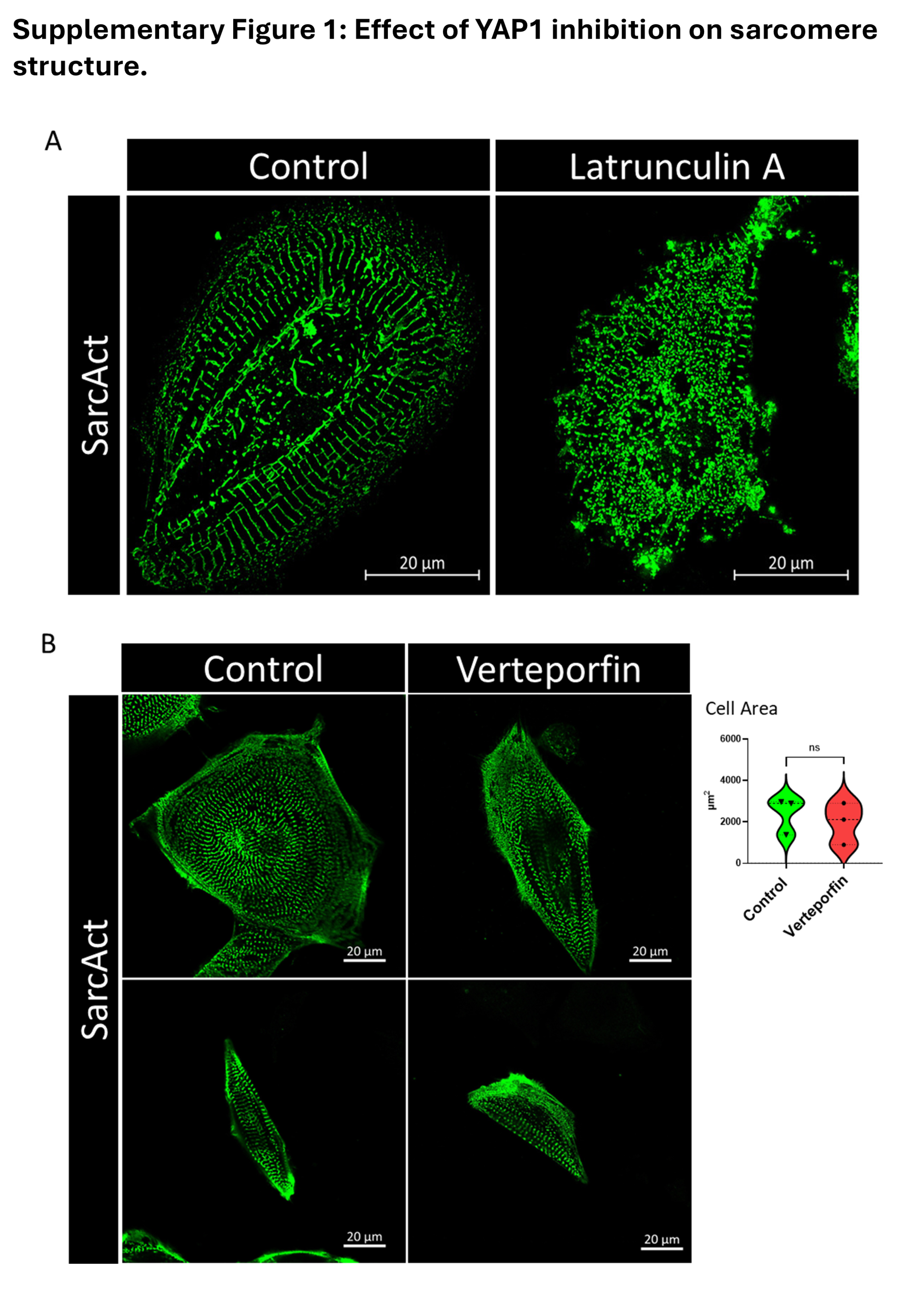

Supplement: Supplementary file 3 — Supplementary Figure 1: Effect of YAP1 inhibition on sarcomere structure. [file 41420_2025_2793_MOESM3_ESM.tif]

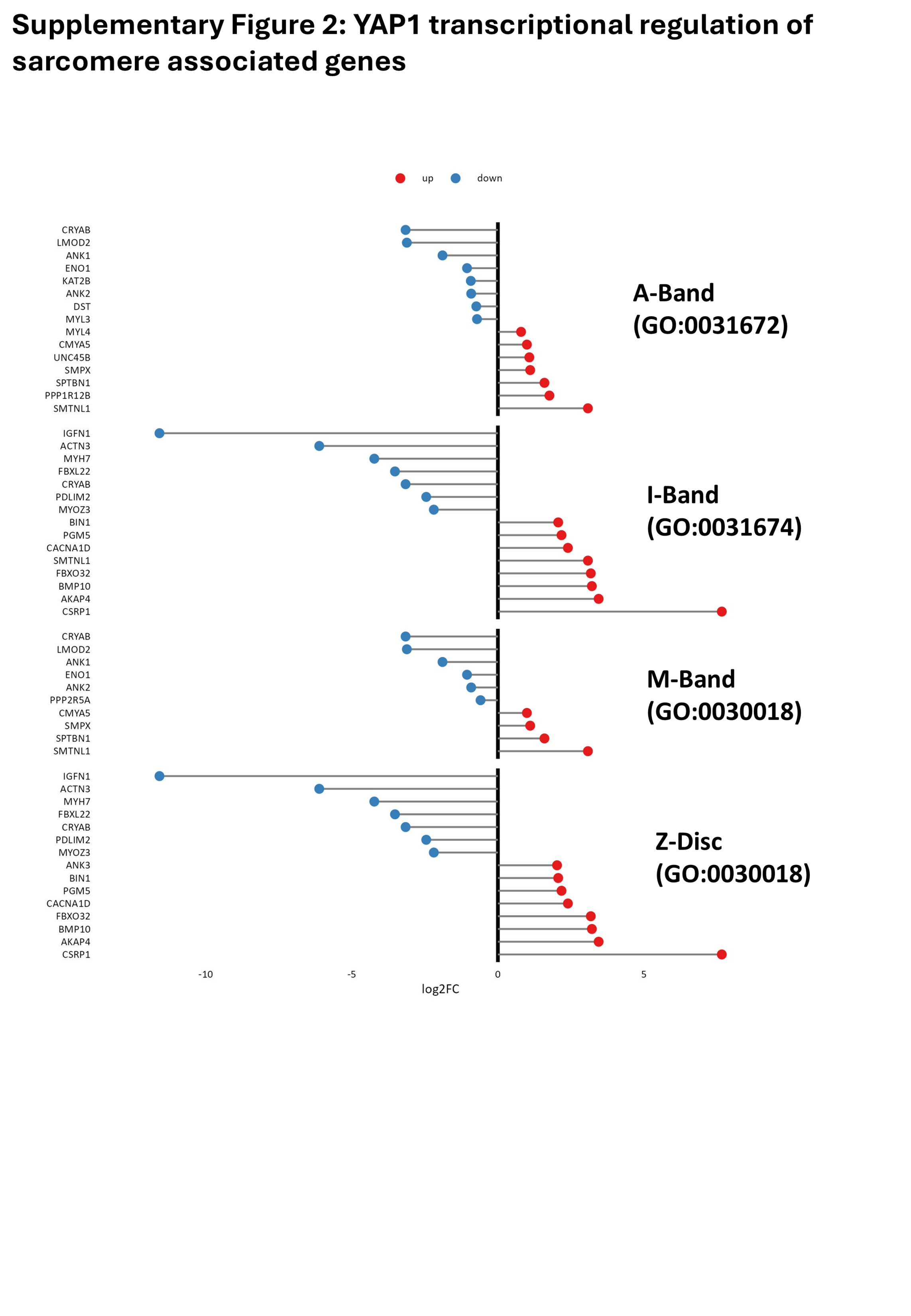

Supplement: Supplementary file 4 — Supplementary Figure 2: YAP1 transcriptional regulation of sarcomere associated genes [file 41420_2025_2793_MOESM4_ESM.tif]

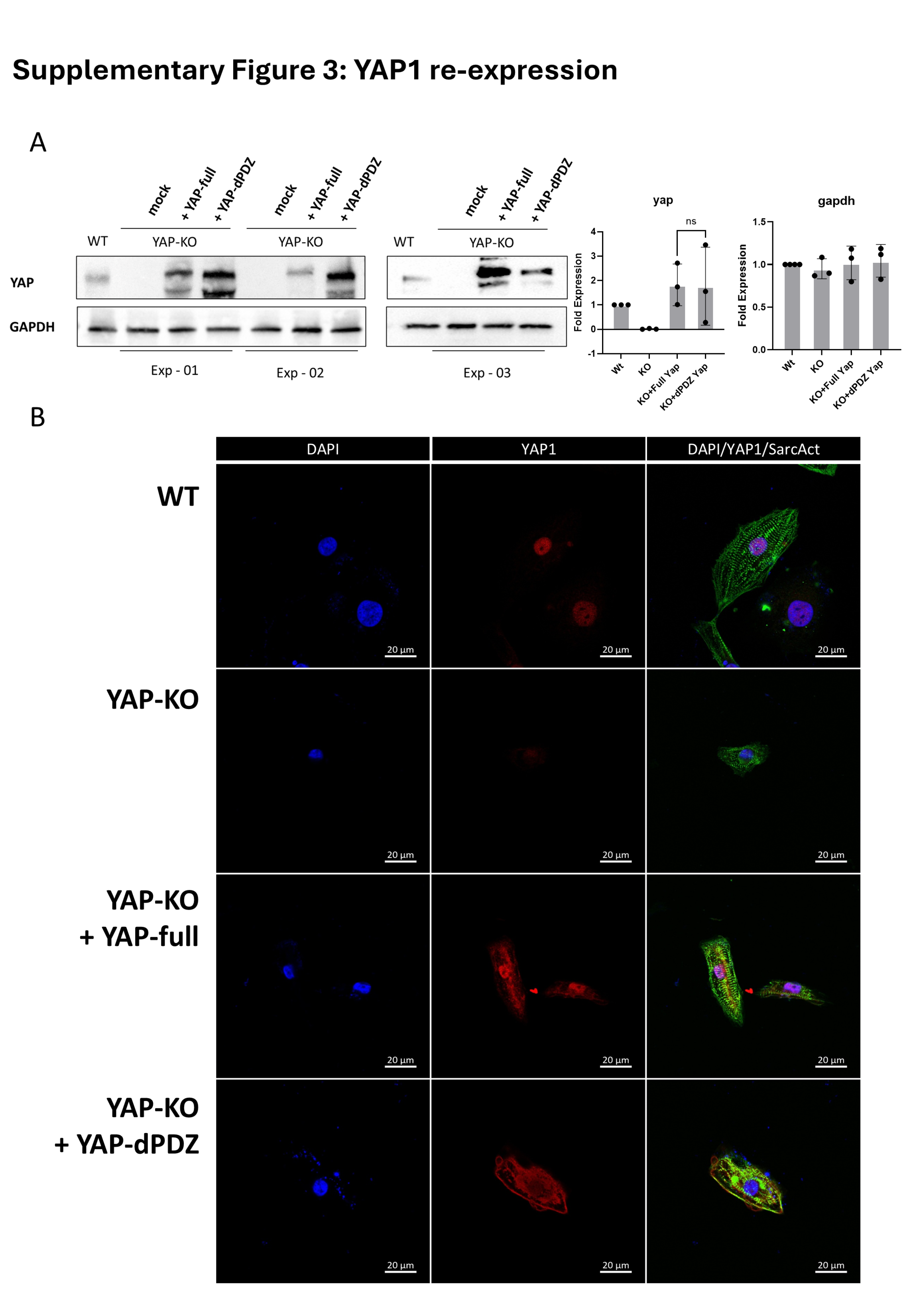

Supplement: Supplementary file 5 — Supplementary Figure 3: YAP1 re-expression [file 41420_2025_2793_MOESM5_ESM.tif]

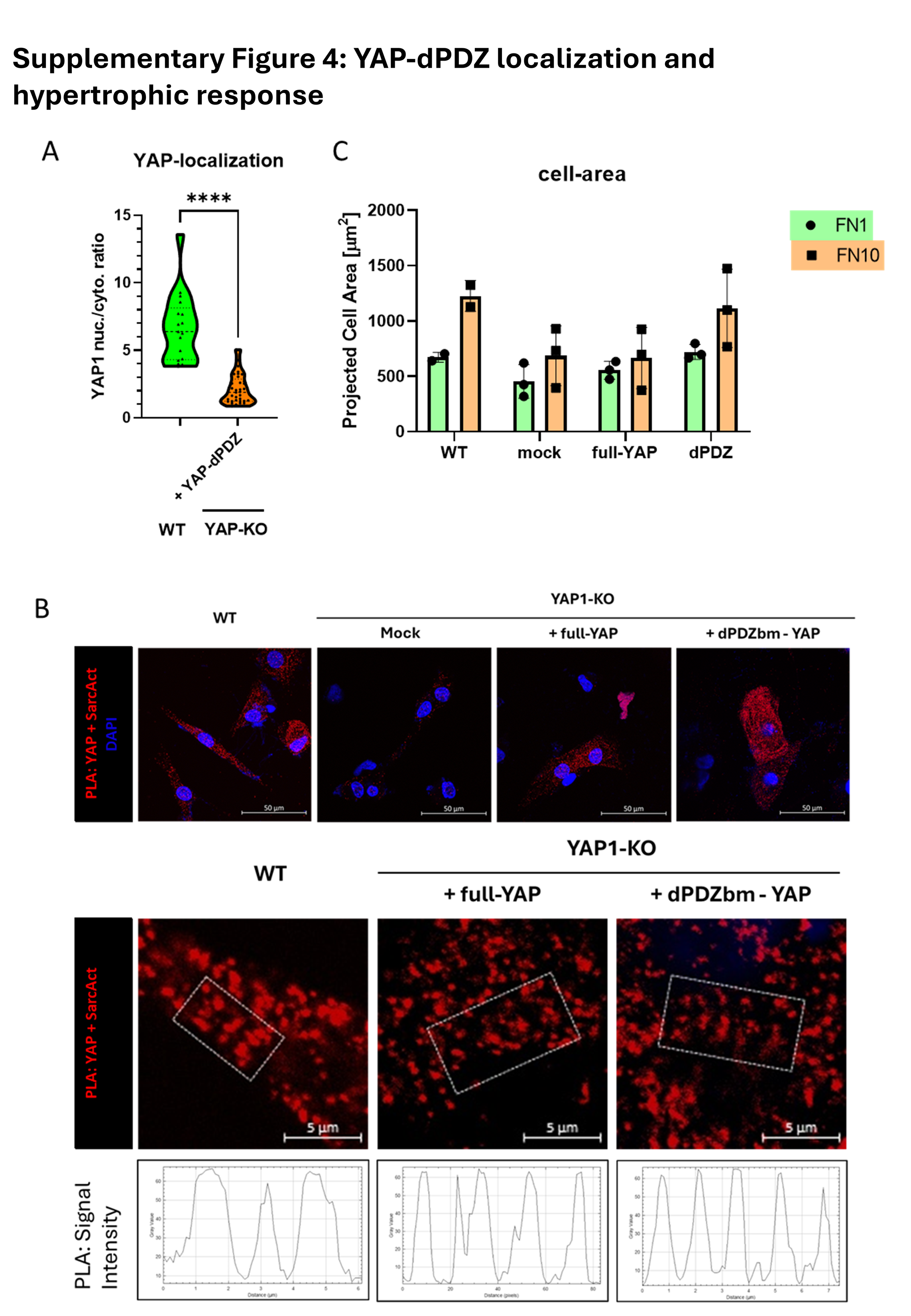

Supplement: Supplementary file 6 — Supplementary Figure 4: YAP-dPDZ localization and hypertrophic response [file 41420_2025_2793_MOESM6_ESM.tif]

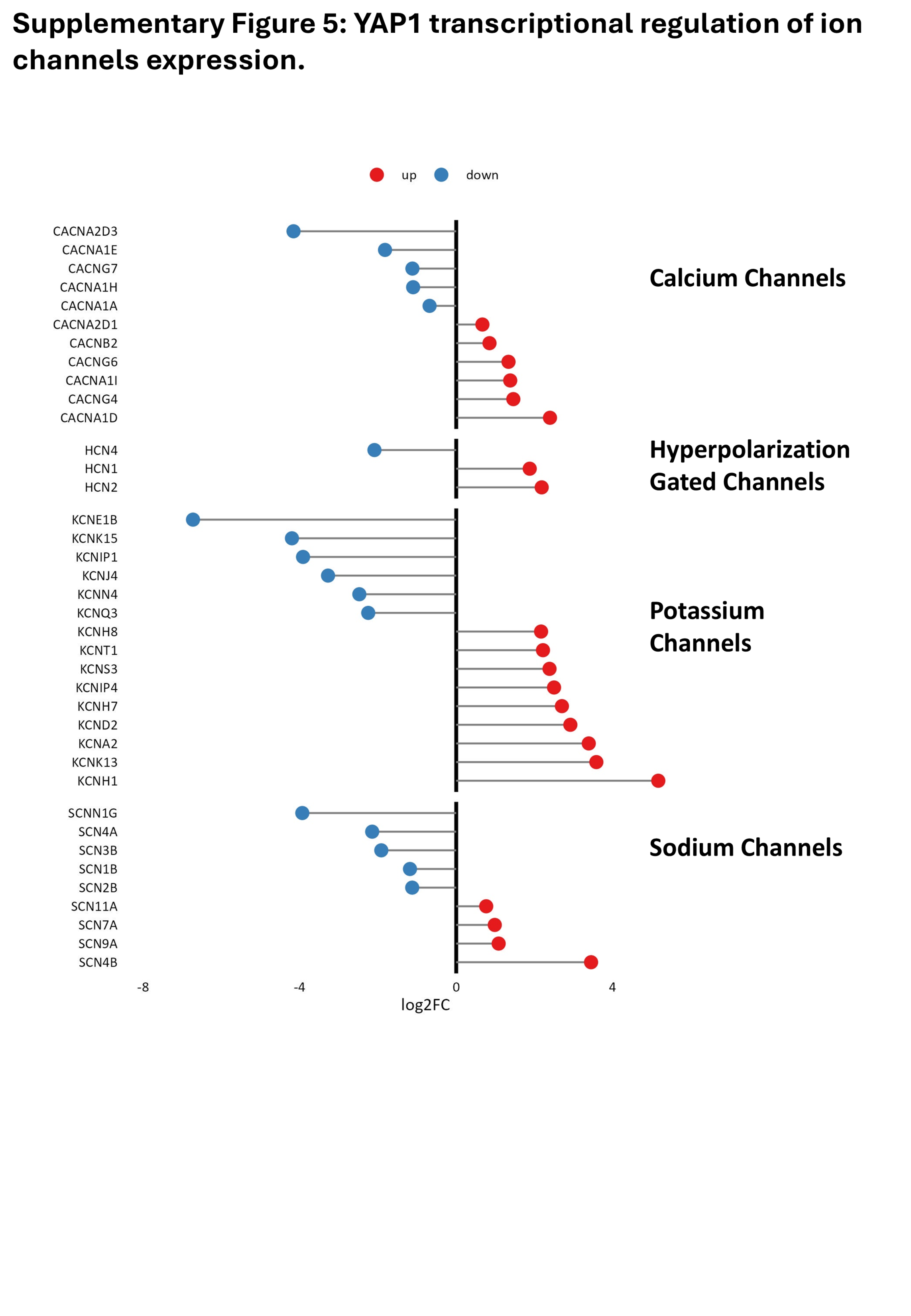

Supplement: Supplementary file 7 — Supplementary Figure 5: YAP1 transcriptional regulation of ion channels expression. [file 41420_2025_2793_MOESM7_ESM.tif]

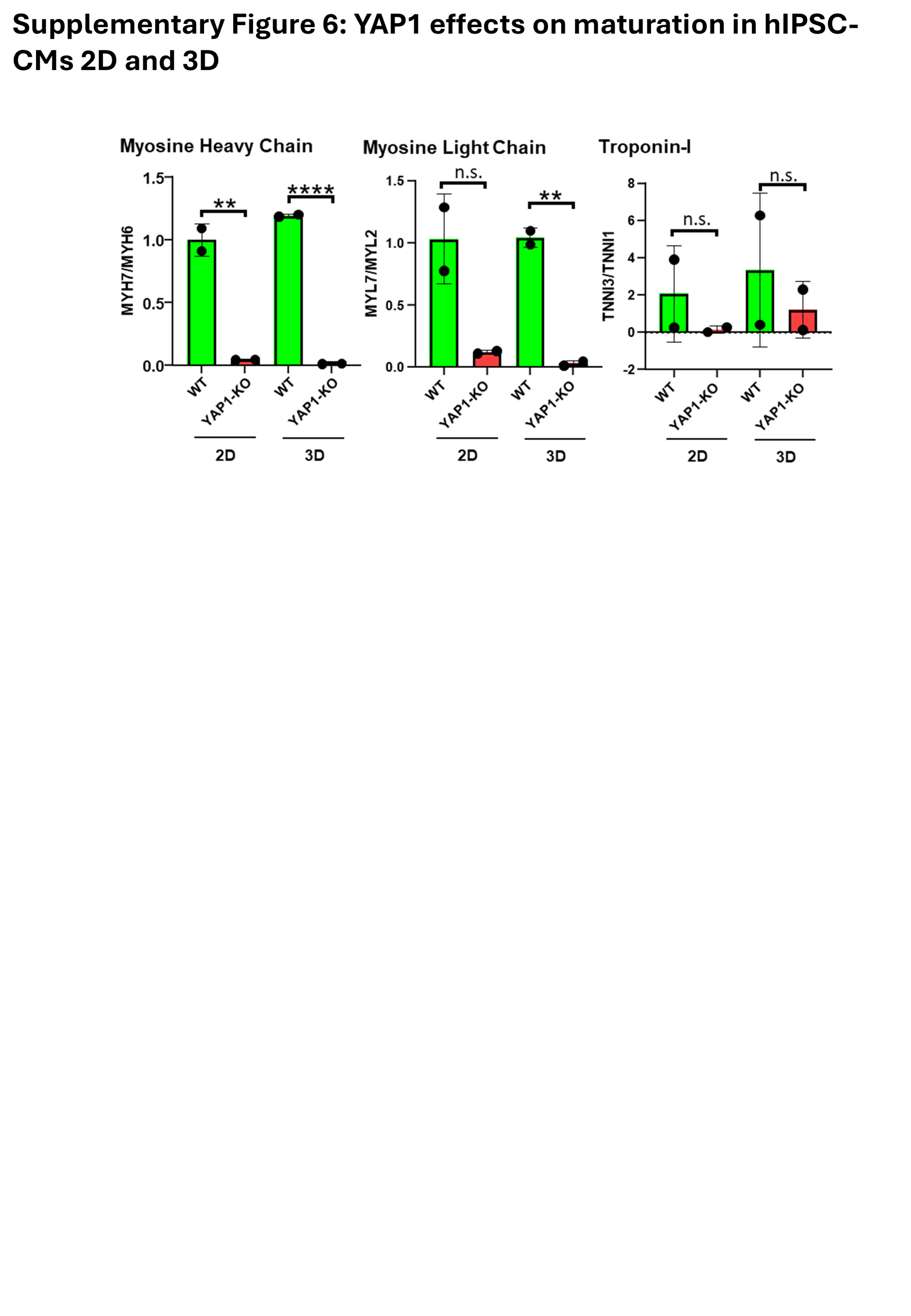

Supplement: Supplementary file 8 — Supplementary Figure 6: YAP1 effects on maturation in hIPSC-CMs 2D and 3D [file 41420_2025_2793_MOESM8_ESM.tif]
